# International Society of Sports Nutrition position stand: diets for body weight reduction

**DOI:** 10.1080/15502783.2026.2740266

**Published:** 2026-10-13

**Authors:** Alan A. Aragon, Bill I. Campbell, Brad J. Schoenfeld, Jeffrey R. Stout, Douglas Kalman, Paul J. Arciero, Shawn M. Arent, Richard B. Kreider, Lem W. Taylor, Laurent Bannock, Robert E.C. Wildman, Chad M. Kerksick, Jose Antonio

**Affiliations:** a Fit Advancement, LLC, Chatsworth, CA, USA; b Exercise Science & Kinesiology Program, University of South Florida, Tampa, FL, USA; c Department of Exercise Science and Recreation, CUNY Lehman College, Bronx, NY, USA; d School of Kinesiology and Rehabilitation Sciences, University of Central Florida, Orlando, FL, USA; e Department of Nutrition, Dr. Kiran C Patel College of Osteopathic Medicine, Nova Southeastern University, Fort Lauderdale, FL, USA; f Human Nutrition and Metabolism Laboratory, Department of Health and Human Physiological Sciences, Skidmore College, Saratoga Springs, NY, USA; g Department of Exercise Science, Arnold School of Public Health, University of South Carolina, Columbia, SC, USA; h Exercise & Sport Nutrition Lab, Department of Kinesiology and Sports Management, Texas A&M University, College Station, TX, USA; i School of Health Professions, Mayborn College of Health Sciences, University of Mary Hardin-Baylor, Belton, TX, USA; j Institute of Performance Nutrition, London, UK; k College of Health and Human Sciences, Kansas State University, Manhattan, KS, USA; l Exercise and Performance Nutrition Laboratory, Lindenwood University, St. Charles, MO, USA; m Department of Health and Human Performance, Nova Southeastern University, Davie, FL, USA

**Keywords:** Fat loss, weight loss, physique, reduced-calorie diets

## Abstract

The International Society of Sports Nutrition (ISSN) has developed an official position on diets for body weight reduction. This critical analysis of the literature is intended to provide a scientific foundation for practitioners, coaches, and trainees regarding the effects of the major diet types in adults who are generally healthy, physically active, and/or athletic, while drawing from clinical research in those with overweight/obesity, when relevant. This position stand does not address acute dehydration-based weight cutting, pharmacotherapy (i.e. GLP-1RAs), or bariatric surgery. The following 15 points constitute the Position Statement of the Society; the Research Committee has approved them.

1) Diets for body weight reduction can be broadly categorized by the magnitude of the energy deficit, linearity of the deficit, and macronutrient composition. These factors need to be tailored to individual preferences, tolerances, and goals.
2) Very-low-energy diets (<800 kcal/day) and low-energy diets (800–1200  kcal/day) produce greater short-term weight loss but lack long-term advantages over less aggressive energy restrictions, such as calorie-controlled diets. These aggressive diets may increase lean mass loss, although adequate protein intake and resistance training may mitigate this risk. Caution is warranted about insufficiencies in vitamins, minerals, fiber, and other nutrients.
3) The evidence at-large shows a lack of differential effects between intermittent fasting variants (alternate-day fasting, time-restricted eating, and twice-weekly fasting) and continuous energy restriction on anthropometric outcomes, although macronutrient quality plays an independent role in optimizing body composition and metabolic health.
4) Intra-day meal frequency (as opposed to total daily nutrition) does not tend to crucially influence body weight and body fat reduction. Meal frequency may be individualized flexibly; no lower or upper limits can universally be prescribed to optimize outcomes. However, in physically active and athletic populations, distributing protein across at least three protein-rich meals (or eating occasions) per day may optimize lean mass retention and performance during sustained energy deficits.
5) Resistance training and sufficient protein intake are indispensable mitigators of lean mass loss in a sustained caloric deficit, regardless of the diet type or variant. Total daily protein is of primary importance; specific distribution of the constituent doses is a secondary concern.
6) For the general population, a daily protein intake of 1.2–1.6 g/kg BW is appropriate for supporting total body weight and fat loss, physical activity, and imparting benefits to musculoskeletal and cardiometabolic health.
7) Daily protein intakes ranging 1.6–2.2 g/kg BW combined with resistance training may optimize muscle anabolism, while higher intakes of 1.9–3.2 g/kg BW can maximize lean mass retention in lean, resistance-trained individuals sustaining hypocaloric conditions.
8) The dietary carbohydrate‒fat proportion can be individualized to personal preference for the goal of weight loss, since the range of ketogenic to low-fat diets performs similarly.
9) Ketogenic diets are effective at bodyweight and body fat reduction, but they also tend to decrease fat-free mass to a greater degree than higher-carbohydrate comparators, particularly in shorter trials where glycogen and total body water shifts can influence estimates.
10) Adaptations to weight loss can be physical, physiological, and/or behavioral, and converge toward reducing energy expenditure to fulfill the body’s drive toward neutralizing disruptions to homeostasis imposed by the caloric deficit.
11) Adaptive thermogenesis is a reduction in resting energy expenditure beyond that predicted by changes in body composition. It can occur in response to hypocaloric conditions and weight loss, but it is not an inevitable response, and it may attenuate substantially upon attainment of weight stability.
12) Metabolic adaptations that reduce energy expenditure, including adaptive thermogenesis, and impair gut microbial diversity, can often be attenuated by strategic dietary and exercise programming that foster nutrient-absorption and partitioning, and metabolic flexibility.
13) A multitude of tactics can be implemented to manage and overcome plateaus. However, plateaus are also opportunities/cues to practice maintaining progress made thus far (the ultimate goal is the maintenance of a plateau).
14) Guidance from an interdisciplinary team of health professionals while maintaining social support is recommended for successful weight management.
15) Learning how to prepare home-cooked meals (and plan meals ahead of time through the day) puts dieters in control of their nutritional fate – and in a better position to succeed in managing their body weight and body composition.

## 1. Background

Globally, obesity prevalence has approximately tripled since 1975, and is projected to reach 25% in 2035 [1]. Obesity prevalence in the United States is estimated to be 40.3%, with no statistically significant difference between men and women [2]. Obesity-related comorbidities are numerous and include coronary heart disease, type 2 diabetes, stroke, and cancer. The health benefits of weight reduction are apparent at even modest degrees [3]. As little as 2%–5% weight loss can improve glycemic control and lower triglyceride levels. Moderate weight loss (5%–10%) can improve blood pressure, decrease joint pain, increase mobility, improve gut microbiome diversity [4], reduce symptoms of depression, and lower cardiovascular mortality risk [3,5]. Substantial weight loss (15%) has resulted in remission of type 2 diabetes [6].

Although net losses in body weight can be sufficient for alleviating obesity-related complications, optimizing this process involves the attenuation of lean mass loss, which characterizes *high-quality weight loss* [7]. A consequence of low-quality weight loss called “collateral fattening” is the physiological drive to recover from lean tissue losses, which amplifies appetite, hyperphagia, and ultimately a rebound in body fat (i.e. “fat overshooting” [8,9]. High-quality weight loss has distinct advantages for athletic performance. Excess fat tissue can increase inertia and impede force production [10], and also lower the power-to-weight ratio [11], thereby compromising agility and exercise performance. The preservation of lean mass can thus preserve energy expenditure, immune function, and proper utilization/partitioning of metabolic fuels.

Throughout this paper, the term “lean mass” is used to denote lean soft tissue, while fat-free mass (FFM) refers to all non-fat tissue (including bone mineral content). These constructs are often used interchangeably in the literature, but changes in lean mass over short interventions may reflect shifts in glycogen and total body water rather than contractile tissue, particularly with ketogenic diets or very low-energy intakes. Interpretation of body composition outcomes should therefore consider the assessment method (e.g. dual X-ray absorptiometry, DXA; bioelectrical impedance analysis, BIA) and intervention duration.

Body mass index (BMI), calculated by dividing weight in kilograms by height in meters squared, is considered useful as a rough anthropometric indicator for disease risk stratification in population-based studies [12]. BMI categories are as follows: underweight (<18.5); normal weight (18.5–24.9); overweight (25–29.9); Class I obesity (30–34.9); Class II obesity (35–39.9); and Class III obesity (≥40). Although BMI has utility as a screening tool at the population level, its utility for assessing health/fitness at the individual level is limited. The critical issue with BMI is that it correlates poorly with body fat percentage (BF%) [13–15].

Arriving at BF% guidelines for the general public is difficult, given the array of methodological limitations and heterogeneity across studies. Nevertheless, body composition standards can alleviate the shortcomings of BMI, and better assess individual status. An earlier publication by Abernathy and Black [16] suggested that a healthy BF% range was 12%–20% for men and 20%–30% for women. Kyle et al. [17] used more rigorous methods (FFM index & body fat mass index) to arrive at a range of 10.8%–21.7% for men, and 21.7%–33.2% for women. Salmon-Gomez et al. [18] outlined a nuanced set of BF% strata, as follows: men: underweight (<10%); normal weight (10%–<20%); overweight (20%–<25%); and obesity (≥25%). Women: underweight (<20%); normal weight (20%–<30%); overweight (30%–<35%); and obesity (≥35%). A relatively recent systematic review and dose-response meta-analysis was conducted by Jayedi et al. [19] that included 35 cohort studies and found a J-shaped association between adiposity and mortality, where the lowest risk of death from all causes was at a BF% of 22 in men and 35 in women. Accounting for metabolic syndrome prevalence, Potter et al. [20] identified BF% thresholds for BMI-defined states of overweight and obesity at 25% and 30% in men, and 36% and 42% in women, respectively. The collective evidence therefore suggests that healthy BF% targets for men and women are approximately 11%–22% and 20%–35%, respectively. These BF% reference ranges are summarized in Table 1.

**Table 1.** Body fat percentage reference values.

Author/Publication	Evidence/Approach	Men: BF%	Women: BF%	Notes
Abernathy & Black [16]	Earlier publication proposing healthy BF% ranges	12%–20%	20%–30%	Earlier, a relatively broad definition of a “healthy” BF% range. Still a reasonable guideline.
Kyle et al. [17]	Used FFM index and body fat mass index to derive body-composition reference ranges	10.8%–21.7%	21.7%–33.2%	More methodologically rigorous approach; ranges are somewhat broader than Abernathy & Black.
Salmon-Gomez et al. [18]	Proposed BF% strata corresponding to weight-status categories	Underweight: < 10% Normal: 10%–<20% Overweight: 20%–<25% Obesity: ≥25%	Underweight: < 20% Normal: 20%–<30% Overweight: 30%–<35% Obesity: ≥35%	Provides nuance via categorical thresholds rather than a single “healthy” range.
Jayedi et al. [19]	Systematic review and dose-response meta-analysis; 35 cohort studies	Lowest all-cause mortality risk at approximately 22%	Lowest all-cause mortality risk at approximately 35%	The authors found a J-shaped association between adiposity and all-cause mortality. These are mortality-risk nadirs, not necessarily recommended BF% targets.
Potter et al. [20]	Cross-sectional NHANES analysis; BF% thresholds derived from MetSyn prevalence corresponding to BMI-defined overweight and obesity	No MetSyn cases: <18% Overweight threshold: 25% Obesity threshold: 30%	No MetSyn cases <30%; Overweight threshold: 36% Obesity threshold: 42%	Proposed outcome-based %BF thresholds to replace BMI-based definitions of overweight and obesity
Collective evidence	Synthesis of the above findings	~11%–22%	~20%–35%	Approximate healthy BF% targets based on the collective evidence.

It is worth reiterating that general guidelines should not be rigidly applied to individuals. Values below these ranges (i.e. in athletes and naturally leaner individuals) can still qualify as healthy. However, while the alleviation of overweight and obesity results in favorable health and performance outcomes, the pursuit of excessive leanness carries its own set of caveats. A sustained state of low energy availability can increase injury risk, exacerbate or foster psychological disorders, and eventually lead to a condition called relative energy deficiency in sport (RED-S), which is characterized by a constellation of pathologies across the immunological, neuroendocrine, musculoskeletal, and cardiovascular systems [21].

The diversity of dietary approaches to weight loss is accompanied by claims of varying veracity. This presents the challenge of distinguishing evidence-based information from falsehood pervading the media landscape. It is of prime importance for clinicians and coaches to understand and apply the state of science related to diets for body weight reduction. For the goal of weight loss, it is axiomatic that hypoenergetic (negative energy balance; caloric deficit) conditions must be sustained [22,23]. The present position stand examines the capabilities and implications of common dietary approaches toward the goal of high-quality weight loss. This position stand focuses on dietary strategies to reduce body weight and fat mass, at the same time maintaining lean mass in adults, who are generally healthy, physically active, and/or athletic, while drawing on overweight/obesity research when the evidence base is primarily clinical. It does not address acute dehydration-based weight cutting, pharmacotherapy, or bariatric surgery. Much of the long-term weight loss literature derives from overweight/obese clinical populations. Extrapolation to lean, athletic, or resistance-trained individuals should be interpreted within that specific context. These clarifications are important for preventing overgeneralization in applied settings.

Finally, protein dosing recommendations in this article that are given on a *per-unit-of-mass* basis (i.e. grams of protein per kilogram) are in reference to “ideal” or normal body weight, since this reflects the context of most of the studies on acute anabolic response. The use of current rather than targeted body weight inevitably varies in appropriateness, depending on individual degrees of adiposity. Practically speaking, for individuals with obesity, the recommendations would tend to overestimate needs based on current bodyweight; thus, using ideal or targeted bodyweight is most prudent for this population. The major dietary approaches discussed in this position stand, along with their defining characteristics, strengths, limitations, and potential applications, are summarized in Table 2.

**Table 2.** Summary of dietary approaches.

Diet	Characteristics	Strengths	Limitations	Potential applications
Very-low-energy diet (VLED)	Formula-based total diet replacement providing ~400–800 kcal/day for 8–16 weeks, followed by gradual food reintroduction.	Produces the most rapid short-term weight loss (typically 10%–15% body weight); improves cardiometabolic risk factors; early rapid loss may enhance long-term success in obesity.	High risk of lean mass and bone mineral loss if not combined with adequate protein/resistance training; weight regain is common after cessation; adverse effects (e.g. constipation, dizziness, gallstones); inappropriate for lean individuals; may result in nutrient insufficiencies; may require medical supervision; severe energy restriction may impair training and adherence.	Severe obesity requiring rapid weight reduction (e.g. preoperative or medically supervised treatment).
Low-energy diet (LED)	Provides ~800–1200 kcal/day, often using partial or total meal replacement.	Substantial weight loss with lower severity than VLED; easier transition to long-term eating.	Lean mass loss remains a concern; long-term outcomes similar to more moderate approaches; may result in nutrient insufficiencies; aggressive energy restriction may impair training and adherence.	Clinical weight management where a large but less aggressive energy deficit is appropriate.
Protein-sparing modified fast (PSMF)	High-protein variant of VLED (typically ~1.5–2.0 g/kg, sometimes higher) designed to preserve lean tissue during severe caloric restriction.	Greater lean mass preservation than conventional VLEDs; rapid fat loss.	Still may require medical supervision; severe energy restriction may impair training and adherence; unsuitable for lean individuals; may result in nutrient insufficiencies.	Individuals with obesity require rapid fat loss while minimizing muscle loss.
Intermittent fasting (IF, general category)	IF alternates fasting and feeding periods; includes alternate-day fasting (ADF), twice-weekly fasting (5:2), and time-restricted eating (TRE). *These IF variants are detailed below.*	Comparable to continuous energy restriction (CER) for weight loss and cardiometabolic improvements, simple rules may improve adherence without calorie counting.	No clinically meaningful superiority to CER when energy intake is matched; lean mass preservation depends on protein intake and resistance training.	Individuals who prefer structured fasting schedules over daily calorie restriction.
Alternate-day fasting (ADF)	Alternates fasting (or ~25% energy intake) with unrestricted feeding days.	May produce slightly greater short-term weight loss than other IF variants.	Greater risk of lean mass loss due to larger intermittent deficits; adherence may be challenging. A 4:3 model (3 fasting days per week) may be a more feasible yet similarly effective alternative to ADF.	Individuals comfortable with full-day fasting who prioritize simplicity.
Twice-weekly fasting (5:2/TWF)	Two low-calorie fasting days each week with habitual intake on the remaining days.	Effective and flexible; similar outcomes to CER.	No consistent advantage over CER for body composition or health outcomes.	Individuals preferring intermittent rather than daily restriction.
Time-restricted eating (TRE)	Restricts daily eating to a 4–10-hour window without necessarily prescribing calorie intake.	Simple implementation; effective ad libitum strategy for reducing energy intake; early TRE tends to show better glucose control.	No independent metabolic advantage beyond caloric deficit in the long-term; effectiveness depends on spontaneous energy reduction.	Individuals preferring meal-timing strategies over calorie tracking.
High-protein diet	Protein intake generally ≥ 1.2–1.6 g/kg BW/day; often 1.6–2.2 g/kg BW or higher during energy restriction or athletic training (lean, resistance-trained individuals can benefit from 1.9–3.1 g/kg BW)	Increases satiety and thermogenesis; improves fat loss while preserving lean mass; supports muscle protein synthesis; safe in healthy individuals across a wide intake range.	Very high intakes may be unnecessary for sedentary individuals; benefits depend on adequate resistance training and total energy balance.	Weight-loss programs, athletes, resistance-trained individuals, older adults, and anyone prioritizing lean mass retention.
Very-low-fat diet (VLFD)	Fat intake typically 10%–20% of total energy.	Can produce meaningful weight loss and improve cardiovascular risk factors.	No long-term fat loss advantage over higher-fat diets; adherence may be difficult; evidence base relatively limited.	Individuals preferring predominantly plant-based, low-fat dietary patterns.
Low-fat diet (LFD)	Fat intake is typically 20%–35% of total energy with higher carbohydrate intake.	Effective for weight loss when calories are restricted; comparable long-term efficacy to LCDs.	No consistent superiority over other macronutrient distributions; adherence may decline over time.	General population seeking a conventional, guideline-based weight-loss diet.
Low-carbohydrate diet (LCD)	Restricts carbohydrate intake (typically 50–150 g/day or ≤45% of energy) without necessarily inducing ketosis.	Greater short-term weight loss than LFDs; may improve glycemic control and satiety.	Weight-loss advantage diminishes beyond 12–24 months; benefits largely disappear when calories and protein are matched.	Individuals preferring carbohydrate restriction, particularly those with obesity or impaired glucose regulation.
Ketogenic diet (KD)	Very-low-carbohydrate diet (≤50 g/day or ≤10% of energy), inducing nutritional ketosis, with high fat and moderate protein intake.	Effective for short-term weight and fat loss; suppresses appetite; increases fat oxidation; may improve glycemic control.	No intrinsic fat-loss advantage when protein and calories are matched; greater lean mass loss than comparator diets; LDL cholesterol often increases; keto-adaptation symptoms common; long-term adherence may be challenging.	Individuals who strongly prefer carbohydrate restriction or require nutritional ketosis, provided lean mass preservation and lipid responses are carefully monitored.

## 2. Methods

ISSN position stands are invited papers on topics that the ISSN editors and Research Committee identify as topics of interest that provide guidance to readers and the profession. Senior JISSN editors and/or the Research Committee identify a lead author or team of authors to perform a comprehensive literature review. The draft is then sent to leading scholars for review and comment. The paper is then revised as a consensus statement and reviewed and approved by the Research Committee and Editors as the official position of the ISSN.

To inform this position stand, a literature search was conducted in PubMed/MEDLINE and Google Scholar (last search: August 7, 2026), using combinations of terms related to energy restriction, intermittent fasting (ADF, TRE, 5:2), protein intake, meal frequency/protein distribution, low-carbohydrate diets, ketogenic diets, low-fat diets, adaptive thermogenesis, and weight loss plateaus. Systematic reviews/meta-analyses and randomized controlled trials were prioritized when available, supplemented by controlled feeding studies and relevant mechanistic work. The reference lists of the included papers were hand-searched for additional citations. The evidence was appraised qualitatively (no formal GRADE process was applied).

Graphic summaries (Figures 1–6) are provided for Sections 3–8. These figures are intended to serve as visual learning tools for distilling key concepts, reinforcing practical take-home points, and conveying the bigger picture among the myriad details.

**Figure 1.**
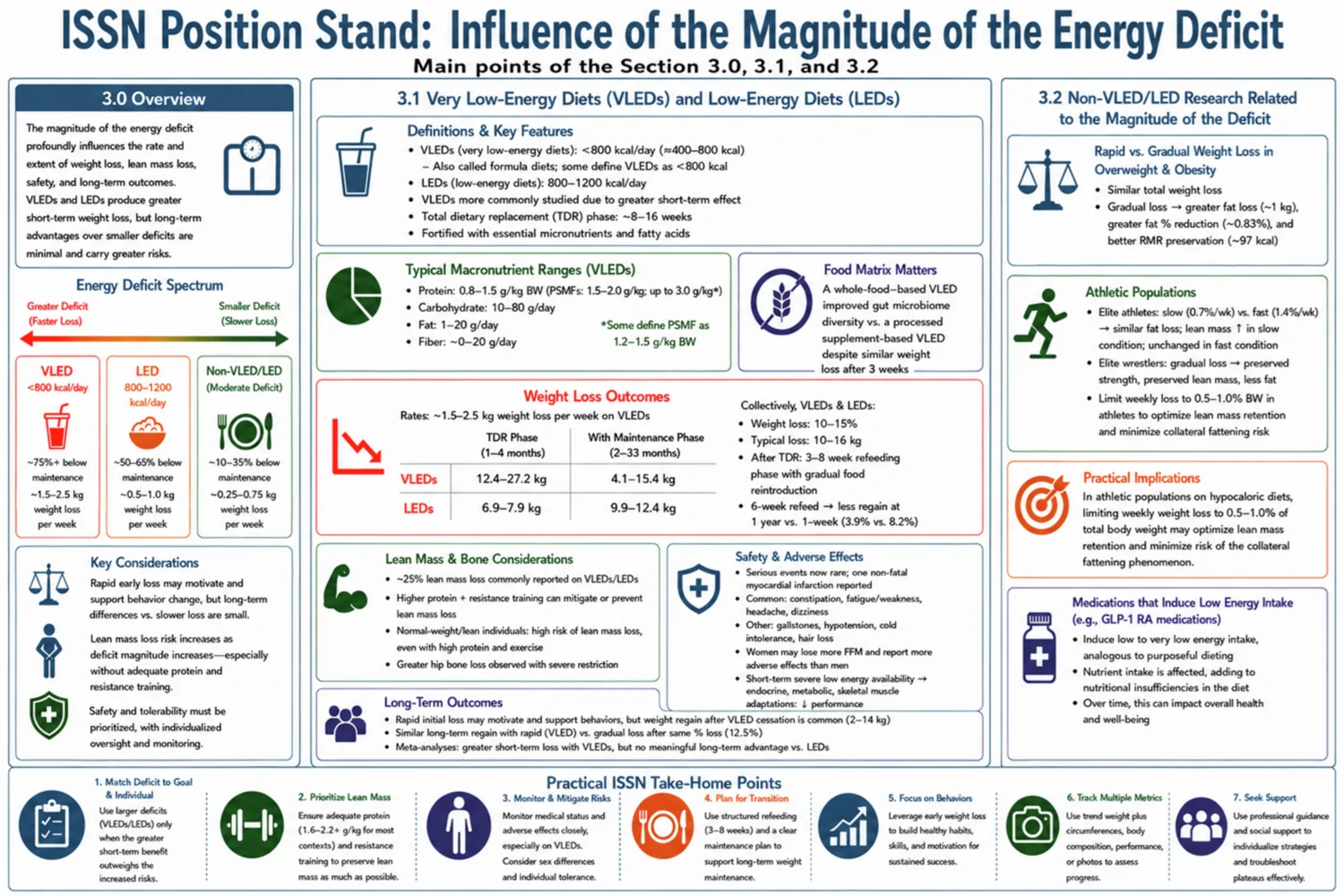
Influence of the magnitude of the energy deficit. Overview of how the magnitude of energy restriction influences the rate and composition of weight loss, safety, and longer-term outcomes, with practical considerations across clinical and athletic populations. *Figure designed with assistance from ChatGPT (OpenAI).*

**Figure 2.**
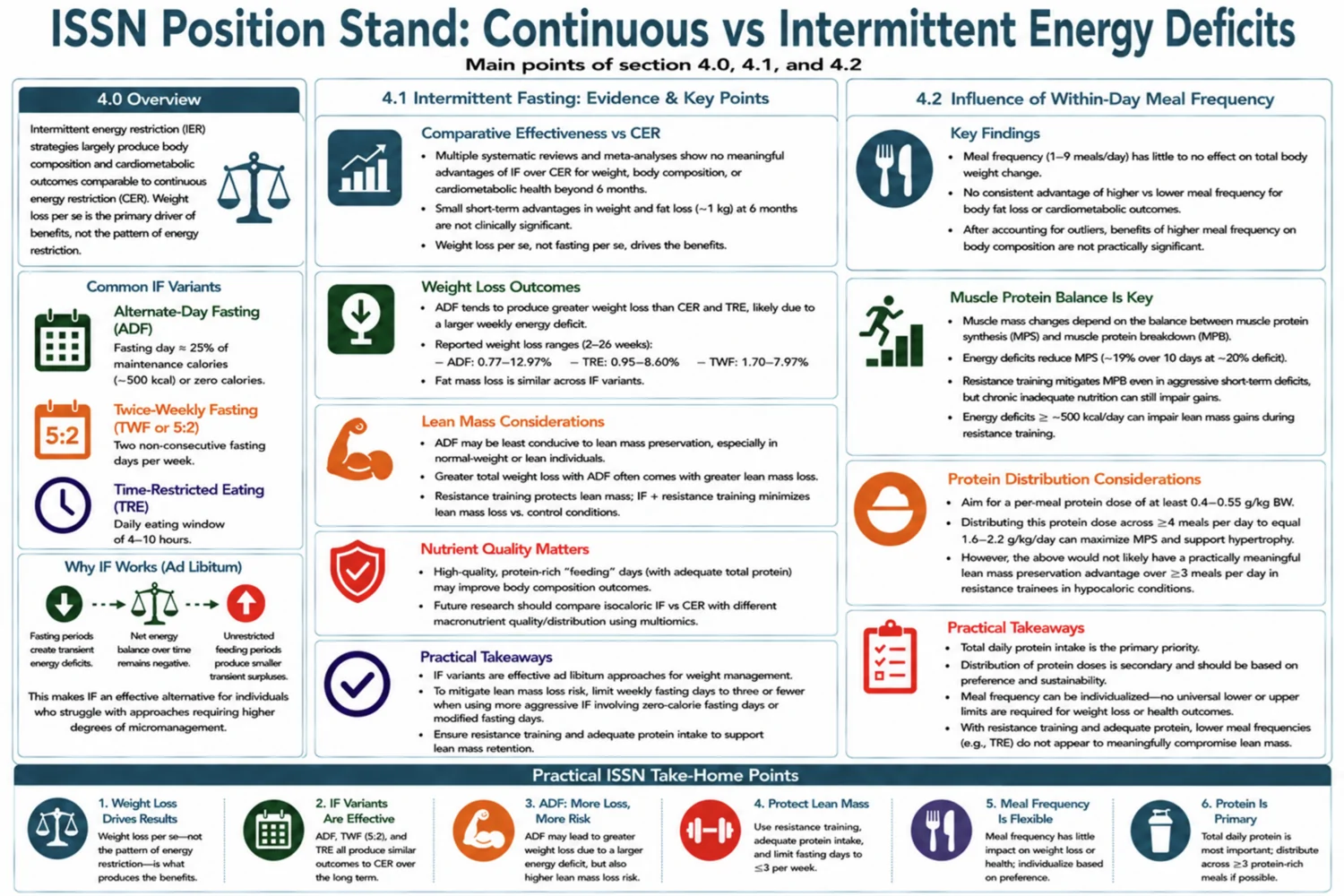
Continuous vs intermittent energy deficits. Overview of the comparative effects of intermittent and continuous energy restriction on weight loss and body composition, with practical considerations for fasting variants, meal frequency, protein distribution, and lean mass preservation. *Figure designed with assistance from ChatGPT (OpenAI).*

**Figure 3.**
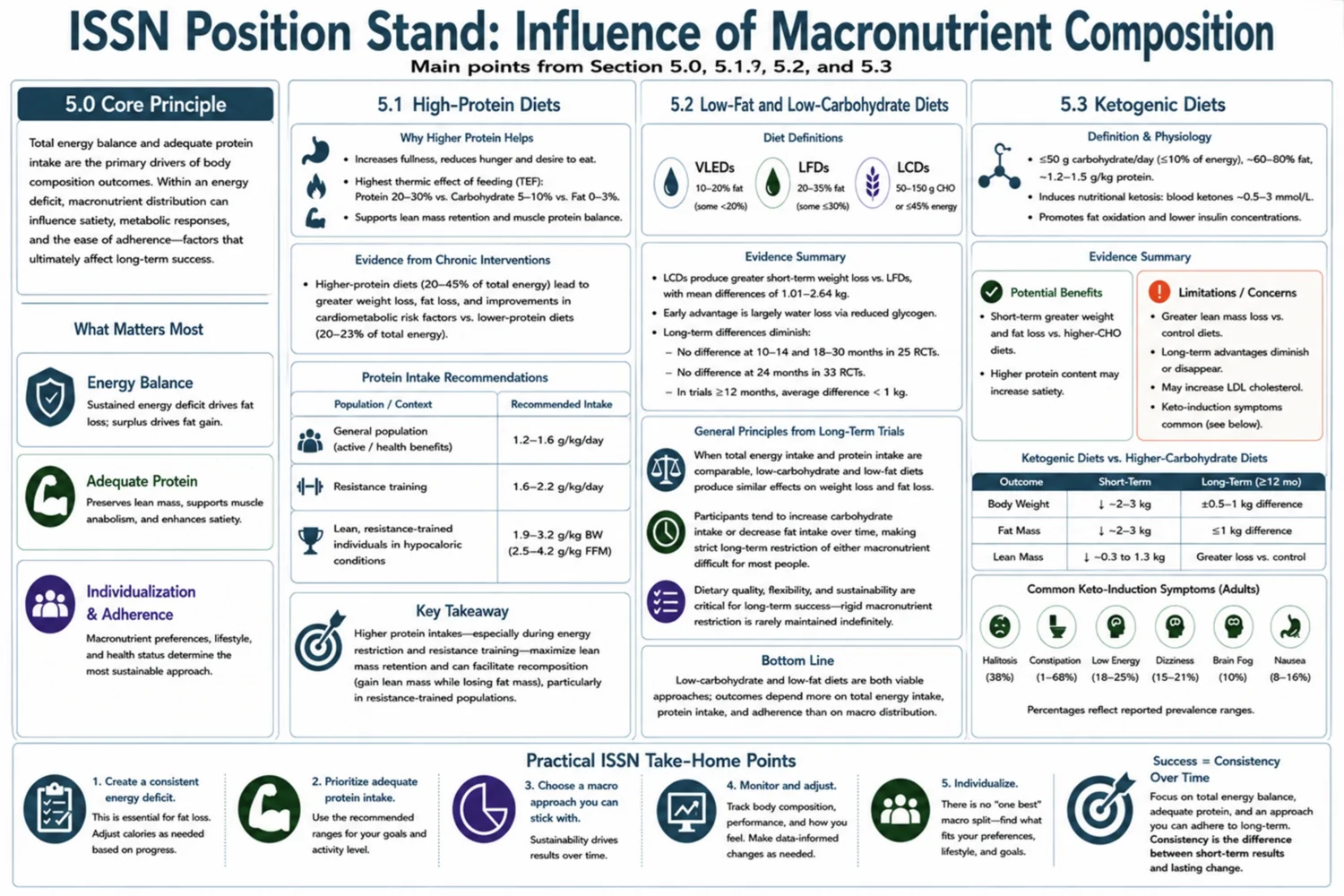
Influence of macronutrient composition. Overview of how protein intake and dietary carbohydrate–fat distribution influence weight loss, body composition, and related outcomes, with consideration of higher-protein, low-fat, low-carbohydrate, and ketogenic diets. *Figure designed with assistance from ChatGPT (OpenAI).*

**Figure 4.**
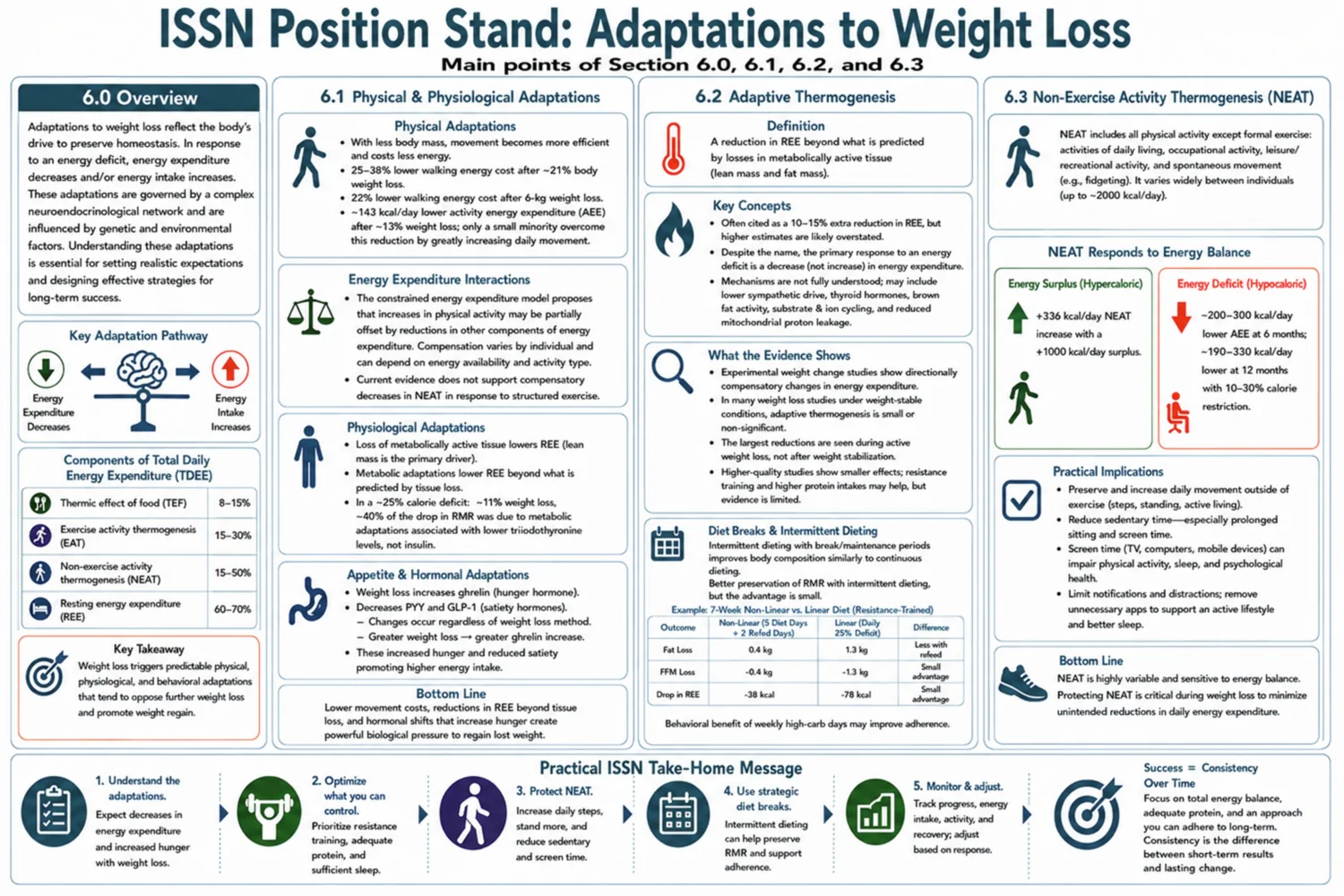
Adaptations to weight loss. Overview of the physical, physiological, and behavioral adaptations to weight loss, including changes in energy expenditure, adaptive thermogenesis, appetite regulation, and non-exercise activity, with practical considerations for mitigating their impact. *Figure designed with assistance from ChatGPT (OpenAI).*

**Figure 5.**
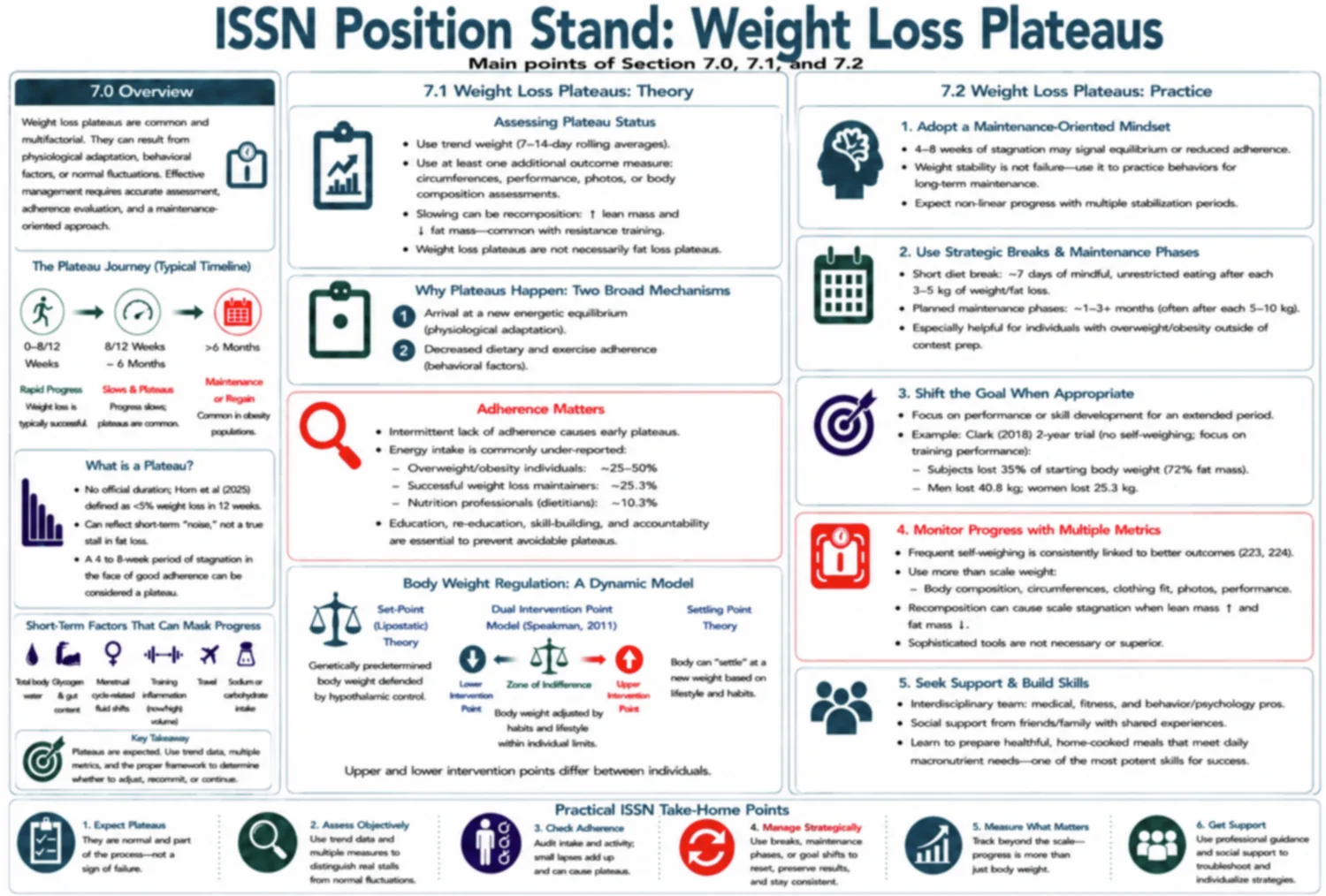
Weight loss plateaus. Overview of the physiological and behavioral factors underlying weight loss plateaus, with practical strategies for assessing progress, managing plateaus, and transitioning between continued weight reduction and weight maintenance. *Figure designed with assistance from ChatGPT (OpenAI).*

**Figure 6.**
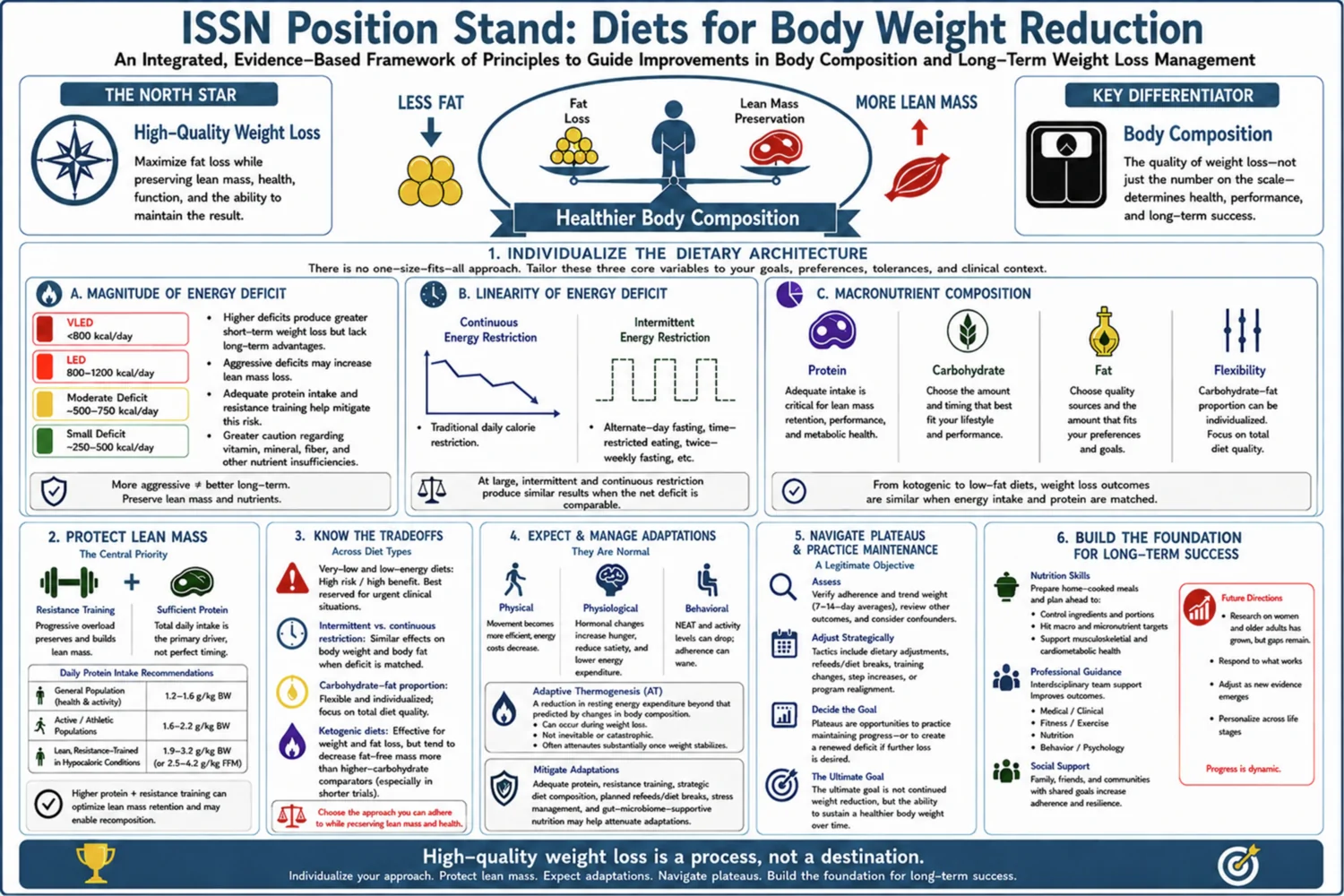
Diets for body weight reduction: position stand summary of principles & practical applications. Integrated overview of evidence-based principles for high-quality weight loss, emphasizing individualized dietary approaches, lean mass preservation, management of adaptations and plateaus, and the behavioral and support strategies underlying long-term weight management. *Figure designed with assistance from ChatGPT (OpenAI).*

## 3. Influence of the magnitude of the energy deficit

### 3.1. Very low-energy diets (VLEDs) and low-energy diets (LEDs)

Diets of the highest hypoenergetic magnitude are termed very-low-energy diets (VLEDs). Also referred to as *formula diets*, VLEDs are commercially produced and contain fewer than 800 kcal, with a lower end of ~400 kcal [24]. Low-energy diets (LEDs) contain 800–1200 kcal/day. It is worth noting that some publications categorize 800-kcal diets as VLEDs, while others classify VLEDs as less than 800 kcals. There is no official consensus on this threshold – and this may be the reason VLEDs and LEDs in the literature are commonly lumped into a single discussion. Of the two types, VLEDs are more prolifically studied – presumably owing to their greater effect on short-term weight loss.

VLEDs are designed to completely replace regular/solid food intake in the initial *total dietary replacement* (TDR) phase for durations ranging approximately 8–16 weeks. Thus, they are fortified with essential micronutrients (vitamins, minerals) and essential fatty acids. The protein content of VLEDs generally ranges from 0.8 to 1.5 g/kg BW. The carbohydrate, fat, and fiber content range from 10 to 80 g, 1–20 g, and nearly none to 20 g, respectively. High-protein variants of VLEDs are referred to as *protein-sparing modified fasts* (PSMFs), with protein content ranging from 1.5 to 2.0 g/kg BW, and as high as 3.0 g/kg [25]. Of note, there seems to be a lack of consensus on the definition of PSMF, since there is also literature describing its protein content as 1.2–1.5 g/kg BW [26–28]. Beyond macronutrient composition, the food matrix may also influence physiological responses to VLEDs. In a recent randomized controlled-feeding trial, Lane et al. [29] found that a VLED composed predominantly of whole-food ingredients increased gut microbiome diversity and richness compared with an isocaloric supplement-based VLED composed primarily of highly processed ingredients, although no between-group differences were observed in anthropometric or other clinical outcomes after 3 weeks.

Weight loss on VLEDs is rapid, with rates ranging from ~1.5 to 2.5 kg per week. This substantial weight loss is expected, given the massive energy deficit imposed by VLEDs, which are typically ~75% (or more) below the daily caloric maintenance requirements of the intended user. Body weight reduction during the TDR phase of VLEDs (1–4 months) ranges from 12.4 to 27.2 kg, and with a maintenance phase (2–33 months), weight loss ranges from 4.1 to 15.4 kg. In LEDs, weight loss during the TDR phase (1–4 months) ranges from 6.9 to 7.9 kg, and with a maintenance phase, weight loss ranges from 9.9 to 12.4 kg. Collectively, VLEDs and LEDs result in weight losses of 10%–15%, with typical losses of 10–16 kg [30]. After the TDR phase, a 3 to 8-week refeeding phase involving the gradual reintroduction of regular foods is typically recommended to assist in weight loss maintenance [24]. Gripeteg et al. [31] found that a 6-week refeeding phase resulted in significantly less weight regain than a 1-week refeeding phase (3.9% vs. 8.2%) at 52 weeks, indicating greater effectiveness of a more prolonged food-reintroduction period.

A 25% lean mass loss as a result of VLEDs and LEDs is commonly reported [32–34]. Illustrating this, a 12-month trial by Seimon et al. [35] compared “moderate” energy restriction (25%–35% energy deficit) with “severe” energy restriction (65%–75% energy deficit for 4 months, followed by 8 months of moderate restriction) in postmenopausal women with obesity. At the 4-month mark, the weight loss in these groups was 17.4 and 7.1 kg, and the lean mass loss was 3.7 and 1.4 kg, respectively. At the 12-month mark, weight loss was 15.3 and 8.4 kg, and lean mass loss was 3.2 and 2.1 kg, respectively. Limitations to these findings are that protein intake (1.0 g/kg) was just slightly above the RDA, and no formal exercise training was engaged during the trial. On a cautionary note, there was a greater loss of total hip bone mineral density in the severe energy-restriction group compared with the moderate energy-restriction group at 12 months.

It should be recognized that lean mass loss during aggressive caloric restriction, especially in subjects with obesity, can be mitigated or prevented altogether via increased protein intake and resistance training [36,37]. A recent systematic review by Lowrie et al. [38] examined the effect of VLEDs on lean mass reduction. Their review included 7 studies, ranging from 2 to 12 months in duration. Among these 7 studies, 4 included resistance training. Among these 4 studies, 2 reported losses in lean mass, while 2 reported gains in lean mass. It is important to note that the latter two studies do not qualify as VLEDs when strictly applying the definition of <800 kcal/day. In fact, the study showing the most robust gain in lean mass (0.5 kg in 12 weeks) involved a 1120 kcal diet that contained 150 g protein [37]. Missing from this meta-analysis is a 12-week trial by Bryner et al. [36], who reported no significant loss of lean mass despite an 800-kcal intake. Also missing from Lowrie et al. meta-analysis is a 12-week trial by Donnelly et al. [39], who reported increased muscle fiber cross-sectional area (despite reduced lean mass) in resistance-training subjects on an 800-kcal diet. Once again, these diets do not qualify as VLEDs under the <800 kcal definition. Nevertheless, they indicate that lean mass retention is possible despite this highly aggressive caloric deficit in untrained subjects with obesity—at least in the relatively short-term.

For normal-weight or lean subjects, VLEDs and LEDs may potentially pose the risk of lean mass loss, regardless of protein and exercise training interventions. For example, a 21-day trial by Pasiakos et al. [40] compared protein intakes of 0.8, 1.6, and 2.4 g/kg in normal-weight subjects who engaged in moderate levels of cardiorespiratory and resistance training while on a 40% caloric deficit diet, which is only approximately half the deficit imposed by a VLED. Lean mass losses (as a percentage of total body weight) were 58%, 30%, and 36%, respectively. Quoting Mustajoki and Pekkarinen [24]: *“VLEDs should never be used by normal-weight subjects because it leads to excessive loss of lean body mass. This warning should be widely communicated.”*

In subjects with obesity, more rapid initial weight loss has been associated with greater long-term weight loss [41–43], conceivably by serving as a motivator that leads to healthy behaviors and habit-learning [41]. This finding has implications for the superiority of VLED and LED use in obese subjects. However, weight regain after VLED cessation is a concern, ranging from 2–14 kg in post-intervention periods ranging from 8 days to 5 years [33]. Furthermore, Purcell et al. [42] reported similar weight regain rates between rapid initial weight loss (VLED) versus gradual weight loss (400–500 kcal deficit) after achieving a reduction of 12.5% of baseline body weight. By the end of the 2.7-year maintenance period, both groups regained 73.6% of their lost body weight. Along these lines, a meta-analysis by Tsai and Wadden [44] examined trials with a follow-up assessment at least 1 year after maximum weight loss, and reported greater short-term weight loss with VLEDs (16.1%) versus LEDs (9.7%), but there was no statistically significant difference in the long-term weight loss between the diet types (6.3% vs 5.0%). A more recent systematic review and meta-analysis by Parretti et al. [45] found that VLEDs combined with a behavioral program produced 3.9 kg greater weight loss at 1 year than a behavioral program alone. This advantage decreased to 1.4 kg at 2 years and 1.3 kg at 38–60 months, with the difference no longer statistically significant at the latter time point. While these differences might be statistically significant, the practical significance of greater weight loss in VLEDs is greatly diminished after 1 year.

Serious adverse effects of VLEDs (including sudden death precipitated by cardiac arrhythmias) were more prevalent in earlier research [33,46]. However, a 17-study systematic review by Sallahewa et al. [47] reported no major adverse events except for one study involving a non-fatal myocardial infarction. The most common complaints are constipation, fatigue/muscle weakness, headache, and dizziness [30]. Additional potential adverse effects include gallstones [48], hypotension [46], cold intolerance, and hair loss [44]. A large, multi-center trial by Christensen et al. [49] (the PREVIEW study) examined the 8-week effects of an 810-kcal LED in 2224 overweight, prediabetic subjects with prediabetes, and found that compared to men, women lost less fat mass (7.09 vs. 9.33 kg) and more FFM (3.17 vs. 1.90 kg). Women also had a higher prevalence of adverse effects than men during and immediately after the trial, including constipation, diarrhea, nausea, dizziness, headache, infection, and hair loss. Gout was the only one of the fifteen adverse effects for which men exhibited a higher prevalence. Jeppesen et al. [50] reported that short-term severe low energy availability was associated with adverse endocrine, metabolic, and skeletal-muscle adaptations and impaired exercise performance.

For individuals on prescription medications for weight loss, such as the GLP-1 RA class of medicines, the effect is an induction of low to very low energy intake, analogous to purposeful dieting. Research demonstrates that, with such caloric restriction, nutrient intake is affected, adding to nutritional insufficiencies in the diet [51], which over time, impacts overall health and well-being for the individual.

### 3.2. Non-VLED/LED research related to the magnitude of the deficit

Casting doubt on the universal superiority of rapid weight loss, a systematic review and meta-analysis by Ashtary-Larky et al. [52] determined a similar magnitude of weight loss between rapid and gradual weight loss in subjects with overweight and obesity. However, gradual weight loss resulted in modestly greater reductions in fat mass (1 kg), body fat percentage (0.83%), and better preservation of resting metabolic rate (97.32 kcal). Another trial in this context by Garthe et al. [53] compared the effects of slow (0.7% per week) versus fast (1.4% per week) weight loss in elite athletes in a mix of sports. The results indicated that fat mass decreased to a statistically similar magnitude in both groups, but lean mass increased in the slow weight loss condition, and remained unchanged in the fast weight loss condition. For elite wrestlers, Miranda et al. [54] found that gradual weight loss was not associated with reductions in isokinetic peak torque and was accompanied by reduced body fat and preserved lean mass. In athletic populations sustaining hypocaloric conditions, limiting weekly rates of weight loss to 0.5%–1.0% of total body weight may optimize the retention of lean mass [55,56], thus potentially minimizing the likelihood of the collateral fattening phenomenon [8]. Finally, of relevance to the present subtopic of non-VLED/LED research are the findings of Unick et al. [43,57], showing that faster initial weight loss is a predictor of long-term weight loss maintenance.

### 3.3. Key findings for the magnitude of the energy deficit

- VLEDs and LEDs produce substantial and rapid weight loss, with VLEDs generally producing greater short-term weight loss than does less aggressive energy restriction. This may provide clinical utility when substantial weight reduction is desired over a relatively short period, particularly in individuals with obesity.
- The greater initial weight loss achieved with VLEDs does not consistently translate into superior long-term weight loss. Weight regain is common following VLEDs, and differences in weight loss compared with less aggressive energy restriction are substantially attenuated with longer-term follow-up.
- More aggressive energy restriction may increase the risk of lean-mass loss, particularly in lean individuals, although adequate protein intake and resistance training can mitigate this risk. Accordingly, the magnitude of energy restriction should be individualized according to baseline body composition, goals, and the ability to support lean-mass retention.

## 4. Continuous vs intermittent energy deficits

### 4.1. Intermittent fasting

Over the past decade, a surge in the popularity of intermittent fasting (IF) among the general public has also been reflected in the growing body of IF-related research publications [58,59]. IF involves purposefully extended periods of fasting (or, more accurately, restricted caloric intake) alternated with feeding periods through the course of the day or the week. The most common variants of IF are alternate-day fasting (ADF), twice-weekly fasting (TWF, also called 5:2), and time-restricted eating (TRE), which constrains the daily feeding window to 4–10 h. Much has been written about the cardiometabolic benefits of IF, including improvements in the lipid profile, oxidative stress, inflammation, glucose metabolism, and mitochondrial dynamics [59]. Of particular relevance to body weight regulation, fasting has the potential to liberate large amounts of fatty acids from adipose tissue and increase the expression of peroxisome proliferator-activated receptor alpha [60], thereby enhancing hepatic beta-oxidation.

However, these benefits are likely the result of weight loss via hypoenergetic conditions, rather than inherent or independent effects of intermittent energy restriction [61]. A recent systematic review and meta-analysis by Siles-Guerrero et al. [62] compared the effect of fasting-based strategies with those of control conditions involving continuous energy restriction (CER) on weight loss and metabolic outcomes. The statistically significant advantages of fasting-based strategies in terms of body weight and fat mass reduction (0.94 and 1.08 kg, respectively) by the 6-month mark were deemed too minor to have clinical significance. In studies extending beyond 6 months, there were no statistically significant differences in body weight, body composition, and markers of cardiometabolic health (i.e. blood lipid profile, blood pressure, and glucose metabolism) between the IF and CER. These findings were echoed in another recent systematic review and meta-analysis by Schroor et al. [63] involving 28 RCTs that examined the effects of ADF, TRE, and TWF. Collectively, these IF variants were not superior to CER for improving anthropometric and cardiometabolic outcomes, reiterating the conclusion that weight loss *per se* may be more important than the type of diet for the beneficial effects of IF. With this overarching principle in mind, we can proceed to discuss the minor/nuanced differences in effects between the IF variants.

The majority of ADF trials involve “fasting” days that consist of approximately 25% of the individual’s maintenance caloric requirements (~500 kcal), and are thus labeled *modified* ADF [58]. Zero-calorie ADF research is scarce, but thus far has shown good long-term tolerability [64] and similar effectiveness as daily caloric restriction [65]. Varady and Gabel [66] reported that ADF results in ~3%–7% body weight reduction in trials ranging from 2 to 6 months. However, a recent network meta-analysis by Pascual et al. [67] reported a wider range of weight loss. In studies ranging 2–26 weeks, the body weight reduction in ADF was 0.77%–12.97%. The weight losses in the TRE and TWF were 0.95%–8.60% and 1.70%–7.97%, respectively. Overall, the IF regimens resulted in similar weight loss to that of the CER. However, ADF tended to promote greater weight loss than CER and TRE. In another recent network meta-analysis, Ma et al. [68] also found superior weight loss with ADF compared to CER, but fat mass changes were similar across the IF variants (ADF, TWF, and TRE). Wang et al. [69] conducted an overview of systematic reviews and meta-analyses (upon which further meta-analysis was conducted), and found no statistically significant differences between ADF and TWF on fat mass or lean mass compared to CER. A recurrent theme in the pooled analyses at-large is a lack of differential effects between IF variants on anthropometric outcomes. In contrast to these findings, Arciero et al. [4,70,71] have shown superior body composition (reduced total body, fat, and visceral fat mass) with TWF compared with CER. Plausible explanations for these differences are likely due to the high-quality, nutrient-dense, protein-pacing “feeding” days combined with the two IF days in the Arciero et al. studies, despite identical overall energy intake levels compared with those of the CER group. Thus, future research should compare isocaloric IF versus CER protocols of varying macronutrient quality and distribution using multiomics to advance our understanding of the impact of nutrient quality on body composition differences.

Lean mass loss is a perennial concern, regardless of dietary approach. The tendency towards greater reductions in total body mass via ADF compared to other IF variants is not necessarily surprising, since it can impose a greater weekly net caloric deficit. Aggressive caloric deficits raise the risk of lean mass loss, and among the IF variants, ADF may be the least conducive to lean mass preservation (especially in normal-weight or lean individuals). Illustrating this point, Templeman et al. [72] compared three conditions: (1) “75:75” – daily energy restriction at 75% of habitual intake (25% deficit); (2) “0:150” – zero-calorie alternate-day fasting with 150% of habitual intake on feeding days, thereby matching the energy deficit of 75:75; and (3) “0:200” – zero-calorie alternate-day fasting with 200% of habitual intake on feeding days, thereby maintaining energy balance. In the 75:75 group, 92% of weight loss was attributable to fat mass, compared with only 46% in the hypocaloric ADF group (0:150), with the remainder attributable to fat-free mass. No fasting-specific cardiometabolic advantages were observed. In contrast, Derron et al. [73] recently reported greater fat loss with ADF than with time-restricted eating (TRE) in adults without obesity; however, the ADF group spontaneously reduced energy intake by approximately 34% versus 15% with TRE, whereas Templeman et al. [72] matched energy deficits between ADF and continuous restriction. Thus, the divergent findings may largely reflect differences in the magnitude of energy restriction rather than an intrinsic advantage of fasting for fat loss. Recently, a 12-month trial by Catenacci et al. [74] reported greater weight loss with a 4:3 model (3 weekly modified fasting days; ~20% of maintenance needs) versus daily caloric restriction in adults with overweight or obesity. The between-group weight loss difference was 2.89 kg in favor of the 4:3 model. Lean mass loss was slightly greater in the 4:3 model (between-group difference: 1.2 kg), but there was no specific resistance training assignment in this trial, and protein intake in both groups was low (15% of total kcal; ~0.6 g/kg). The 4:3 model could potentially represent a more feasible alternative to ADF while still retaining its aggressive energy deficit – granted that resistance training and sufficient protein are provided.

It is notable that collectively, ADF [75] and TWF [76] trials resulted in a mean reduction of ~2 kg of lean mass at the 12-week point. A systematic review and meta-analysis by Ashtary-Larky et al. [77] included 8 trials ranging from 4 to 12 weeks, examining the effect of resistance training on IF regimens (2 ADF, 6 TRE trials). Relative to controls, the mean body weight and body fat reductions via IF were 2.08 and 1.36 kg, respectively. The change in lean mass (−0.27 kg) was not statistically significant compared to control conditions. The latter finding underscores the lean mass-protective effect of resistance training.

Although IF variants largely lack anthropometric or clinical advantages over CER, each of the IF variants has consistently shown effectiveness as an *ad libitum* approach [58]. The transient energy deficits imposed by fasting periods (whether intra-day or intra-week) tend to outpace the transient energy surpluses imposed by unrestricted feeding periods. The preservation of net hypoenegetic balance – despite the absence of meticulous dietary tracking – renders IF a viable alternative for individuals whose adherence is compromised using approaches requiring higher degrees of micromanagement.

### 4.2 Influence of within-day meal frequency

Meal frequency (how often/many meals/snacks are ingested/day) is worthy of discussion within the present context since it involves varying “fasting” lengths within the course of a day. Traditional lore suggests that a grazing pattern (frequent, small meals) is necessary to *stoke the metabolism* or increase energy expenditure [78]. However, this hypothesis has been repeatedly refuted in tightly controlled chamber calorimeter studies examining 24-hour energy expenditure of widely disparate frequencies, ranging 2–7 meals daily [79,80]. Longitudinal studies have largely failed to detect a consistent advantage of higher versus lower meal frequencies for body weight management. A meta-analysis by Schoenfeld et al. [81] included 15 studies with meal frequencies ranging 1–9 meals/day, and durations ranging 2–52 weeks. Meal frequency did not statistically affect total body weight change. Higher meal frequencies were associated with greater losses of fat mass and retention of lean mass. However, sensitivity analysis revealed that the removal of a single, 2-week study in boxers [82] eliminated the statistical effect of meal frequency on changes in body composition, leading to the conclusion that the small difference in magnitude of effect between meal frequencies has limited practical significance. More recently, a 16-trial systematic review and meta-analysis by Blazey et al. [83] found no statistically significant advantage of eating in a high-frequency (≥4 meals/day) or low-frequency (≤3 meals/day) dietary pattern for improving cardiometabolic health, including body weight and body fat reduction.

In light of the aforementioned findings, nuance is needed in the discussion of meal frequency for optimizing weight loss outcomes. The protein feeding distribution (across the meals/snacks/day) can potentially influence lean mass preservation. Net muscle protein balance – that is, the cumulative difference over time between muscle protein synthesis (MPS) and muscle protein breakdown (MPB) determines increase, decrease, or maintenance of muscle mass [84]. Periods of hypoenergetic balance can compromise muscle preservation by mitigating MPS and/or promoting MPB. Pasiakos et al. [85] reported a 19% decrease in MPS during 10 days of moderate caloric deficit (20% below maintenance). Resistance training has been shown to mitigate MPB during an aggressive (40%) short-term (10-day) caloric deficit [86]. However, this protective effect is apparently not durable; training adaptations may still be compromised by chronically inadequate nutritional support, even at moderate deficits. Corroborating this point, a systematic review and meta-analysis by Murphy and Koehler [87] examined RCTs with a minimum 3-week duration; and found that energy deficits at or beyond 500 kcal/day can impair lean mass gains in subjects undergoing resistance training. However, a caveat to these findings is that the majority of studies did not report actual protein intake, and there was high heterogeneity of protein intake assignment across studies.

Given the catabolic nature of hypocaloric conditions, it is warranted to strive to maximize the anabolic effect of each meal to preserve muscle protein balance. Schoenfeld and Aragon [88] proposed that MPS (and in turn, muscle hypertrophy) is maximized via protein dosed at 0.4–0.55 g/kg per meal over the course of at least 4 meals to a total of 1.6–2.2 g/kg/day. Along these lines, Arciero et al. [89,90] have repeatedly demonstrated that a high daily protein intake (>1.4 g/kg) consumed across 4–6 meals per day outperforms control diets of lower protein and meal frequency for fat mass reduction and lean mass preservation in hypocaloric conditions. More recent trials by Arciero et al. [71,91] found that the latter approach (high-protein/high-frequency) combined with 1–2 weekly modified fasting days (~500 kcal/day) was superior to the conventional control diets for improving body composition in a caloric deficit, most likely driven by enhanced gut microbiome diversity and metabolomic signatures [4]. These findings support an independent role of macronutrient quality beyond overall caloric intake as a primary metabolic advantage during caloric restriction. In physically active and athletic populations, distributing protein across 3 or more protein-rich meals (or eating occasions) per day may support lean mass retention and performance during sustained energy deficits [55,56]. It should be noted that many trials in this area combine higher protein intakes, structured exercise, and other co-interventions, limiting causal attribution to meal frequency per se.

### 4.3. Key findings of continuous versus intermittent energy deficits

- Intermittent fasting generally produces body composition and cardiometabolic outcomes comparable to continuous energy restriction. Some individual trials report greater weight or fat loss, particularly with high-protein protocols, but these effects cannot be attributed to fasting per se and are not consistently observed.
- Alternate-day fasting may produce somewhat greater weight loss, potentially owing to a larger net energy deficit, but may also increase lean mass loss. Resistance training and purposefully limiting weekly fasting days to three or fewer can mitigate this risk.
- Meal frequency per se appears to have little effect on weight loss or cardiometabolic outcomes. The upper and lower “limits” of meal frequency can largely be dictated by personal preference and individual adherence capability. In athletic populations, adequate protein intake distributed across multiple protein-rich meals (3 or more) may optimize lean mass retention (or gain) during energy restriction, particularly with resistance training.
- Although the findings in this area are mixed, muscle retention in hypocaloric conditions is unlikely to be meaningfully compromised with lower meal frequencies, as seen in TRE trials involving resistance training. It bears emphasis that total daily protein is of primary importance, with distribution of the constituent doses being of secondary importance (and bet left to individual preference and long-term sustainability).

## 5. Influence of macronutrient composition

### 5.1. High-protein diets

In the context of high-quality weight loss, the importance of adequate or optimal dietary protein intake cannot be overstated [92]. Aside from the provision of amino acids for preserving or increasing the net muscle protein balance, protein has the highest thermic effect of feeding (TEF). This can be viewed as the energetic cost of processing within the body; protein is the most metabolically expensive macronutrient. The TEF of protein, carbohydrate, and fat is approximately 20%–30%, 5%–10%, and 0%–3%, respectively [93]. In acute interventions, protein consistently increases fullness, and decreases hunger and the desire to eat [94]. These subjective effects are accompanied by objective mechanisms, including decreased ghrelin, and increased cholecystokinin and glucagon-like peptide-1 (GLP-1) [95,96]. In chronic interventions, higher-protein conditions consistently outperform lower-protein comparators for weight loss-related outcomes. Illustrating this, a large systematic review and meta-analysis by Vogtschmidt et al. [97], which included 54 RCTs found that higher-protein diets (20%–45% of total energy) resulted in greater weight loss, fat loss, and improvements in cardiometabolic risk factors (systolic blood pressure, total cholesterol, triglycerides, and insulin) than lower-protein comparator diets (20%–23% of total energy).

The current RDA for protein (0.8 g/kg/day), while generally sufficient to meet the basic protein requirements of healthy, sedentary adults, is unlikely to be optimal – and may be insufficient for preserving or maximizing lean mass and optimizing body composition – in many populations and physiological states, particularly during exercise training, aging, and energy restriction [98–104]. For the general population, a daily protein intake of 1.2–1.6 g/kg is appropriate for supporting physical activity and imparting preventive/clinical benefits to musculoskeletal, gut microbiome, and cardiometabolic health [4,92,101,105]. For the first time in its history, the Dietary Guidelines for Americans (2025–2030) explicitly recommended a daily protein intake of 1.2–1.6 g/kg for adults, providing further support for protein intakes above the longstanding RDA [106]. Protein intakes ranging from 1.6 to 2.2 g/kg may optimize muscle anabolism when combined with resistance training [102,107–114]. The joint position stand of the Academy of Nutrition and Dietetics, Dietitians of Canada, and American College of Sports Medicine recommends 1.2–2.0 g/kg [103] for physically active and athletic populations. The most recent position stand of the International Society of Sports Nutrition recommends 1.4–2.0 g/kg, with intakes beyond this range potentially benefiting resistance trainees in hypocaloric conditions [98].

Indeed, the evidence has accumulated in favor of protein intakes significantly beyond the higher end of the traditionally recommended ranges. For athletic populations in hypocaloric conditions, there is considerable evidence that even higher protein intakes can benefit or optimize weight loss outcomes. A 6-study systematic review by Helms et al. [115] reported that a daily protein intake of 2.3–3.1 g/kg FFM was most effective for body composition outcomes in lean, resistance-trained individuals undergoing energy restriction. A more recent systematic review and meta-regression by Refalo et al. [116] revisited Helms et al.’s research question and included an additional decade of studies (29 in total), with analyses of non-obese, resistance-trained individuals in hypocaloric conditions. A daily protein intake of 1.9–3.2 g/kg BW, or 2.5–4.2 g/kg FFM, was associated with greater retention of fat-free mass. It is worth emphasizing that the upper end of these ranges is primarily supported in lean, resistance-trained populations and may not apply to individuals with higher body fat percentages or non-training populations.

Recomposition is a relatively novel term denoting the simultaneous gain of lean mass and loss of fat mass. Along similar lines to the findings of Helms et al. [115] and Refalo et al. [116], Barakat et al. [117] reported that a protein intake of 2.6–3.5 g/kg FFM may increase the likelihood and magnitude of recomposition in resistance-trained subjects. A salient example of apparently protein-driven recomposition was by Antonio et al. [118], who compared the effects of normal/habitual protein intake (2.3 g/kg) versus high protein intake (3.4 g/kg) on body composition and performance in resistance-trained subjects. The 8-week trial involved a periodized, progressive resistance training program with sessions occurring 5 days/week. Despite the higher-protein group reporting a 374-kcal increase from baseline, fat loss-dominant recomposition occurred. Both groups gained 1.5 kg of lean mass, but the high-protein group lost more fat mass (1.6 vs 0.3 kg in the control group). Strength and exercise performance improved in both groups, with no statistically significant differences between the groups. Subsequently, a 4-week trial by Longland et al. [119] compared a protein intake of 1.2 vs 2.4 g/kg in overweight and obese subjects in hypocaloric conditions (40% below maintenance caloric needs). The lower-protein group’s lean mass was unchanged, and the fat mass was reduced (3.5 kg). In the higher protein group, fat mass reduced to an even greater degree (4.8 kg), while gaining lean mass (1.2 g/kg). A recent 10-week trial by Vargas-Molina et al. [120] compared high-protein diets (2.5 g/kg) at either maintenance/eucaloric balance or a slight (10%) caloric deficit in resistance-trained subjects. In the hypocaloric group, fat mass decreased (2.94 kg) to a greater degree than that in the eucaloric group (1.41 kg), while both groups gained similar amounts of lean mass (1.04 kg and 0.97 kg, respectively).

These intriguing effects of very high protein intakes are not anomalous; Antonio et al. [121–124] conducted several other trials that consistently showed non-adipogenic effects in resistance trainees assigned additional energy intake (~400–800 kcal) when consumed as protein (2.7–4.4 g/kg). Potential mechanisms of these results are not fully understood, but may include increased satiety, which drives lower intakes of non-protein macronutrients, increased energy expenditure through exercise- and non-exercise-mediated pathways, decreased de novo lipogenesis, and under-reporting of actual protein intakes, or other factors [104].

It is worth noting that traditional concerns with high protein intakes (i.e. adverse effects on kidney, liver, and bone health) lack evidentiary support in healthy populations [123–128]. On a final note, the available evidence does not indicate sex-specific differences in the MPS response to protein feeding. Smith et al. [129] reported a lack of sex-based differences in mixed MPS during a fasted or a hyperinsulinemic-hyperaminoacidemic state in young and middle-aged men and women. Subsequently, West et al. [130] found no sex-based differences in post-exercise myofibrillar MPS in the fasted or protein-fed state.

### 5.2. Low-fat and low-carbohydrate diets

Carbohydrate and fat will be discussed in tandem since varying proportions of these macronutrients characterize the dietary subtypes in question. Very-low-fat diets (VLFDs) have been defined as those containing 10–20% of total energy from fat [131], although some literature designates the upper cut-off below 20%. The data on VLFDs are limited, largely consisting of vegan and vegetarian diets that aggressively restrict fat intake [104]. Despite their capability to facilitate statistically significant weight loss [132], VLFDs lack significant fat loss advantages compared to higher-fat diets, particularly in the long-term [133–135]. Low-fat diets (LFDs) can be defined as those containing 20%–35% of total energy from fat [131], although some literature assigns a 30% upper-end [136]. Low-carbohydrate diets (LCDs) are defined as those containing 50–150 g carbohydrate, or ≤45% of total energy. Ketogenic diets (KDs) are defined as having a maximum carbohydrate content of 50 g (or ≤10% of total energy) [137], and will be discussed independently of LCDs owing to their unique differences.

Several systematic reviews and meta-analyses over the past decade have examined the effects of LCDs, unanimously showing superior weight loss to LFDs [136,138–145], with mean differences ranging 1.01–2.64 kg. However, much of the early weight loss advantage of carbohydrate restriction appears to be from water loss via reduced glycogen storage, with minimal differences in fat loss compared to a non-ketogenic diet [146]. Furthermore, the weight loss advantage of LCDs appears to diminish in the long-term. In a systematic review and meta-analysis of 25 RCTs, Silverii et al. [143] reported that, compared to control diets, LCDs statistically reduced body weight at 3–4 and 6–8 months, but the difference was no longer statistically significant at 10–14 and 18–30 months. Furthermore, in a systematic review and meta-analysis of 33 RCTs, Lei et al. [142] found that LCDs statistically reduced body weight at 6–23 months (mean difference of −1.33 kg), but no statistically significant difference was observed at 24 months. Continuing these findings, a large (61-RCT) systematic review by Naude et al. [147] reported that in trials lasting 3 to < 12 months, the average difference in weight loss between LCDs and control diets was 1 kg, and in trials lasting 12 months or more, the average difference was less than 1 kg.

A noteworthy long-term (12-month) trial in free-living subjects is Gardner et al. DIETFITS study [135], which investigated the effects of aggressive restriction of carbohydrate or fat, using a flexible model that allowed gradual increases in these nutrients in subjects with overweight and obesity. In the first 8 weeks of the study, carbohydrate intake was limited to 20 g/day in one group, while the other group’s fat intake was limited to 20 g/day. From that point, the subjects were encouraged to keep their respective intakes of carbohydrate and fat as low as possible, but were still allowed to increase their daily intakes by 5–15 g/week, as needed. Subjects in both groups were educated on the selection of healthy foods. At 12 months, the low-carbohydrate and low-fat groups showed moderate reductions in body weight (5.29 and 5.99 kg, respectively), with no statistically significant between-group differences. The reductions in body fat content were not statistically significant between the groups (1.97% and 2.15%, respectively). Importantly, there were no statistical between-group differences in protein and total caloric intake. Subjects in both groups were unable to maintain their original (20 g) fat and carbohydrate intake assignments during the initial 8 weeks. Carbohydrate intakes at 12 months in the carbohydrate-restricted and fat-restricted groups were 132.4 g and 212.9 g, respectively. Fat intakes at 12 months were 86.2 g and 57.3 g, respectively.

### 5.3. Ketogenic diets

Unlike the subjective definitions of previous LCDs, KDs can be identified with objective biomarker data. Although there is no single or standard definition, ketogenic diets typically restrict carbohydrate to a daily maximum of 50 g (or ≤10% of total energy), fat ranges ~60%–80% of total energy, and protein is ~1.2–1.5 g/kg BW [104]. KDs induce a state of *ketosis* – a shift in metabolic substrate use, whereby ketones (acetoacetate, acetone, and beta-hydroxybutyrate) are produced as an alternative fuel source to glucose [148]. Carbohydrate (or total energy) restriction can raise circulating ketone levels to a range of ~0.5–3 mmol/L [104]. Ketosis (also referred to as *nutritional ketosis*) is a relatively benign state distinct from *ketoacidosis*, which is the pathological and potentially life-threatening overproduction of ketones in the absence of exogenous insulin [149]. Fasting or severe energy restriction can also induce ketosis [150]. During fasting, the blood ketone concentration (ketonemia) reaches maximal levels within 3 days, plateauing thereafter [151]. With a KD, ketonemia is maximal within approximately 2 weeks [152].

Ketosis has been regarded as a unique and favorable metabolic state, due to lower plasma insulin concentrations and preferential use of fat as a primary fuel source [153]. Indeed, fat oxidation rates are elevated in KDs [154]. Increased fat oxidation (indicated via lowered respiratory exchange ratio) combined with a reduced dependence on glycogen has been coined a state of *ketoadaptation* [104]. Fat oxidation increases via the KD reaches maximal levels and plateaus within the first week of the diet [152]. Increased fat oxidation via a KD is the combined result of increased dietary fatty acid intake, which in turn elevates the circulating free fatty acids and intramyocellular lipid content and utilization. The KD’s preferential use of fat for fuel is mainly a reflection of the high fat proportion of the diet. The protein content of the KD is generally advised to be moderate to preserve a state of ketosis by minimizing gluconeogenesis. However, protein intakes as high as 1.7–2.2 g/kg have resulted in targeted levels of ketosis, with blood ketone levels ranging 0.7–1.8 mmol/L [155–157].

Reduced insulin secretion and circulating insulin concentrations (and concomitant increase in lipolysis) are another commonly proposed mechanism underpinning the body weight reduction effectiveness of the KD. This is the core premise of the *carbohydrate-insulin model of obesity* (CIM), which implicates carbohydrate as the primary insulinogenic agent, and thus the macronutrient to be minimized in the pursuit of body weight loss or prevention of weight gain [158,159]. In light of CIM, KD has been proposed as the ideal dietary solution. However, this idea lacks support from controlled, isocaloric, protein-equated diet comparisons of disparate carbohydrate‒fat proportions, which consistently show no statistically significant fat loss differences between the KD and LFD groups [104,135,148,160,161]. Hall and Guo [160] conducted a meta-analysis of 32 controlled feeding studies, strictly limited to comparisons where protein and energy intake were equated between conditions. No statistically significant differences in weight loss or fat loss were detected, despite carbohydrate proportions ranging 1%–83% and dietary fat ranging 4%–84%.

In studies that do not rigorously control protein and energy intake between participant groups, KDs consistently outperform the higher-carbohydrate/lower-fat comparator diet for body weight and fat reduction. However, KDs also tend to result in greater losses of lean mass compared to control diets. A 13-study systematic review by Coleman et al. [162] reported greater body weight reduction (2.7 vs. 0.3 kg) and body fat reduction (2.3 vs. 0.3 kg) in KDs compared to control diets in physically active subjects. Fat-free mass decreased by 0.3 kg in KD, but increased by 0.7 kg in the control diets. A 13-RCT systematic review and meta-analysis by Ashtary-Larky et al. [163] limited to resistance trainees found significant advantages of KDs over control diets for reductions in body weight (3.67 kg) and fat mass (2.21 kg). However, fat-free mass was reduced by 1.26 kg compared to controls. A subsequent 18-RCT meta-analysis by Koerich et al. [164] found that in athletes and trained subjects, KD resulted in statistically significant reductions in body weight (2.4 kg) and fat mass (2.4 kg) losses, but also greater lean mass losses (0.8 kg) compared to control diets in trials ranging from 4 days to 12 weeks. The authors attributed the weight/fat loss advantages to the greater satiating effect of the higher protein content of KDs.

Lean mass retention challenges of the KD have been proposed to be alleviated by periodic carbohydrate “refeeds,” but available data thus far have not supported this idea. Kysel et al. [165] conducted an 8-week trial comparing the effects of a cyclical ketogenic diet (CKD) involving weekend carb-ups (8–10 g/kg of lean mass on 2 days per week), versus a linear, high-carbohydrate (55%), low-fat (30%), moderately hypocaloric “reduction” diet (RD) in resistance-trained men. The protein concentration was set at 1.6 g/kg in the CKD (carb-up days decreased protein to 15% of total energy) and 15% of the total energy in the RD. Body weight and body fat reductions were statistically significant within both groups. The differences in fat loss between CKD and RD did not reach statistical significance, but the differences (1.9 and 4.0 kg, respectively) favored RD. Lean mass loss was statistically greater in CKD (−1.8 and −0.4 kg, respectively). These findings serve to strengthen the larger body of evidence that KDs excel in fat mass reduction, but are suboptimal for lean mass preservation [166].

The commonly observed advantage of KDs for body weight reduction tends to lack significance in the long term. A 13-RCT meta-analysis by Bueno et al. [167] compared KDs with LFDs in trials lasting a minimum of 12 months. Although KD resulted in statistically greater weight loss than LFD, the absolute difference was relatively minor (0.91 kg) and was no longer apparent at 24 months. It is possible that diminishing adherence is responsible for the progressive lack of difference. Along similar lines, a meta-analysis by Hashimoto et al. [141] found that in >12-month studies, KDs did not result in statistically greater weight loss (0.44 kg), but fat loss was statistically significant (0.97 kg). The authors acknowledged the possibility that KD’s fat loss advantage was protein-mediated, since protein intakes in LCDs and KDs tended to be higher than those of control diets.

Potential adverse effects of KDs warrant consideration. Recent umbrella reviews (quantitative reviews containing multiple meta-analyses) have reported that KDs tend to increase low-density lipoprotein (LDL) cholesterol [168,169]. A solution for this issue would be to modify the food selection of the KD toward a Mediterranean-type model. This would involve moderating/minimizing fatty red meat, processed meat, and butter – and replacing a significant proportion of these foods with unsaturated fat sources such as olive oil, nuts, seeds, and fatty fish, while maintaining high fiber intake from vegetables and fruits [170]. A recent scoping review by Skartun et al. [171] reported that common, transient symptoms experienced during *keto-induction* (transition from a standard, high-carbohydrate diet to nutritional ketosis) in adult populations include the following: halitosis (38%), constipation (1%–68%), liver function abnormalities (24%), feelings of decreased energy (18%–25%), light-headedness/dizziness (15%–21%), skin rash (13%–21%), muscle cramps (3%–37%), headache (8%–25%), nausea (8%–16%), diarrhea (2%–23%), and brain fog/reduced cognitive performance (10%).

### 5.4. Key findings for macronutrient composition

- Higher-protein diets improve weight loss outcomes by enhancing satiety, increasing the thermic effect of feeding, and supporting lean mass retention. Higher protein intakes (particularly during energy restriction) may further optimize body composition and facilitate recomposition – the simultaneous gain of lean mass and loss of fat mass – in resistance-trained populations.
- When energy intake and protein intake are comparable, low-carbohydrate and low-fat diets produce similar effects on weight loss and fat loss, indicating that macronutrient distribution is less important than sustained energy restriction and dietary adherence.
- Ketogenic diets may provide modest advantages for weight and fat loss compared to higher-carbohydrate comparator diets, but these benefits tend to diminish over time, and may be accompanied by greater lean mass loss. This highlights the primary importance of adequate protein intake and appropriate total caloric targets (regardless of carbohydrate‒fat proportion, which should be individualized).

## 6. Adaptations to weight loss

### 6.1. Physical & physiological adaptations

Adaptations to weight loss can be viewed as adjustments in response to changes in physical and physiological circumstances. These adaptations manifest as reductions in energy expenditure and/or increases in energy intake, and are rooted in the perpetual drive toward the preservation of homeostasis. Individual responses to homeostatic disruption are influenced by a mix of environmental and genetic factors. Adaptations to weight loss (imposed by changes in diet and/or physical activity) are governed by a complex neuroendocrinological network that maintains a bidirectional relationship with a wide range of bodily tissues and organ systems, which can ultimately influence eating behavior and energetic balance [172–175]. This section covers the physical, physiological, and behavioral adaptations to weight loss resulting in decreased energy expenditure. The 4 components of total daily energy expenditure (TDEE) and their proportional contributions are as follows [104]:

1) Thermic effect of food (TEF): 8%–15%
2) Exercise activity thermogenesis (EAT): 15%–30%
3) Non-exercise activity thermogenesis (NEAT): 15%–50%
4) Resting energy expenditure (REE): 60%–70%

Physical adaptations to weight loss refer to the increased efficiency and lower energetic cost of movement (in both exercise and non-exercise activity) in a body with less mass than before [176]. For example, Foster et al. [177] reported a 25%–38% reduction in the energetic cost of walking in subjects who lost 21% of their body weight (20.9 kg). Along similar lines, Muller et al. [178] reported a 22% reduction in the energy cost of walking after a 6-kg reduction in body weight. Moreover, Bonomi et al. [179] found that 13% weight loss decreased activity energy expenditure (AEE) by ~143 kcal. Only 5 of the 66 subjects overcame the weight loss-mediated reduction in AEE, which required reducing sedentary time by 2 h per day, and increasing the duration of actively standing (50 min), walking (30 min), and bicycling (5 min).

Beyond reductions in the energetic cost of movement following weight loss, changes in total daily energy expenditure may also reflect interactions among its individual components. The constrained energy expenditure model proposes that increases in physical activity do not necessarily produce proportional increases in total daily energy expenditure; additional energy costs may be partially offset by reductions in other components of expenditure [180]. This *compensation* model contrasts with the traditional *additive* model, which assumes that exercise-related energy expenditure contributes directly to total expenditure. Compensation varies considerably between individuals and may be influenced by energy availability and physical activity dose/type. Pontzer and Trexler [181] reported that the compensation or ‘constraint’ of energy expenditure is reduced with resistance training but pronounced during combined aerobic exercise and dietary restriction. Importantly, the constrained energy expenditure model does not imply that exercise fails to increase energy expenditure or improve health outcomes. Rather, it highlights that the relationship between physical activity and total daily energy expenditure is dynamic and subject to physiological regulation. The current evidence challenges the presumption that exercise constrains or causes compensatory decreases in NEAT. An 11-study systematic review and meta-analysis by Fedewa et al. [182] found that no statistically or practically meaningful change in NEAT occurs in response to structured exercise.

Physiological adaptations to weight loss include changes in metabolically active tissue mass, hormonal changes in appetite regulation, and adaptive thermogenesis (to be discussed in its own subsection). Lean mass (lean soft tissue), often conflated with fat-free mass, is the most significant determinant of resting energy expenditure (REE), accounting for at least 60%–80% of the interindividual variance in REE [183,184]. It should be noted that the traditional emphasis on muscle mass changes that are tightly correlated with changes in RMR is not without dispute. Skeletal muscle is a subcomponent of lean mass, accounting for ~50% of total lean mass, but contributing to only ~30% of REE [185]. Skeletal muscle is energetically dynamic; when used, it can substantially contribute to total energy expenditure. However, at rest, its energy expenditure is relatively minor (~13 kcal/kg/day) compared to other organs, such as the liver, brain, heart, and kidneys, which are 15–30 times more metabolically active at rest than skeletal muscle is [186]. Martin et al. [187] conducted a secondary analysis of the CALERIE study [188]. Body composition and metabolic changes were examined over 12 months of caloric restriction (25% deficit) in 109 subjects. As a result of ~11% weight loss (8 kg), the RMR was reduced by ~100 kcal (~7%) in healthy normal weight and overweight subjects. The intriguing finding was that only 60% of the reduction in RMR (60 kcal/day) could be explained by losses of metabolically active tissues (lean mass and fat mass), while the remaining 40% of RMR reduction (40 kcal/day) was due to metabolic adaptations – which were correlated with reductions in triiodothyronine, leptin, and insulin.

In addition to reductions in energy expenditure, weight loss can alter appetite, priming behavior toward increases in (or impaired efforts to reduce) energy intake. A systematic review and meta-analysis by Jin et al. [189] examined appetite-related gut hormone changes after weight loss. An average weight loss of 6%–7% was observed across studies, and this loss was associated with increased ghrelin and decreased peptide YY (PYY) GLP-1. These alterations in appetite-regulatory hormone levels occurred at similar magnitude regardless of the method of weight loss (caloric restriction, exercise, or a combination). Ghrelin is commonly called “the hunger hormone” owing to its well-established orexigenic (appetite-stimulating) function [190]. Greater weight loss was associated with greater increases in ghrelin levels. PYY and GLP-1 are anorexigenic (appetite-suppressing) hormones with shared mechanisms of action: delaying gastric emptying and exerting vagal and central nervous system effects [191]. GLP-1 has the additional property of functioning as an incretin by stimulating insulin production in response to postprandial glucose elevation. Ultimately, the weight loss-mediated increase in ghrelin and a decrease in PYY and GLP-1 sets the stage for increased hunger and decreased satiety, reflecting the body’s perpetual homeostatic drive.

### 6.2. Adaptive thermogenesis

Adaptive thermogenesis (AT) is an intriguing and important physiological adaptation. AT has been defined as a greater-than-predicted change in energy expenditure in response to changes in energy balance [192]. In the context of dieting to improve body weight or composition, AT can be defined as a reduction in REE beyond what can be predicted by losses in metabolically active tissue (lean mass and fat mass) [193]. AT is commonly cited as a 10%–15% reduction beyond predicted reductions in REE [194], but the current evidence shows that this may be a significant overestimation, especially in weight-stable conditions [195,196].

AT was originally referred to as the thermoregulatory defensive response to decreases in core body temperature during cold exposure [197]. As denoted by the term “thermogenesis,” an autonomic increase in heat production/energy expenditure in cold environments occurs via shivering and non-shivering means—with the latter attributed primarily to increased brown fat activity. The term “adaptive thermogenesis” has since been used to denote reductions in energy expenditure as a result of hypocaloric conditions and body weight reduction. This shift in nomenclature is potentially misleading. The primary metabolic adaptation to hypoenergetic balance is a decrease (not an increase) in energy expenditure, hence the proposal in a previous publication to re-name this phenomenon *adaptive thermoreduction* [104]. The mechanisms underlying AT in the hypocaloric context are poorly understood, but are presumed to include decreases in a range of physiological processes, including sympathetic drive, thyroid hormone secretion, brown fat activity, substrate cycling, ion cycling, and mitochondrial proton leakage [197,198].

A classic investigation by Leibel et al. [199] demonstrated that experimentally induced changes in body weight elicit substantial, directionally compensatory adaptations in energy expenditure: maintenance of a body weight ≥10% below baseline was associated with a reduction in total energy expenditure of approximately 6–8 kcal/kg fat-free mass/day, whereas a 10% increase in body weight increased energy expenditure by approximately 8–9 kcal/kg fat-free mass/day. These adaptations occurred in both individuals with and without obesity and involved changes in resting and nonresting energy expenditure, supporting the concept that human energy balance is subject to physiological regulation that can oppose sustained departures from an individual’s customary body weight.

In recent literature involving weight loss effects, AT has often been interchanged with the term, *metabolic adaptation*. This conservation of energy has been called “hibernation mode” in the literature [193]. In the public domain, AT has been called “starvation mode,” and has been blamed for the failure of weight loss due to a purported critical degree of metabolic slowing. This presumption has received reinforcement from the findings of Fothergill et al. [200], who examined the metabolic effects of substantial weight loss in competitors in the televised Biggest Loser competition. Weight loss at the end of the 30-week competition averaged 58.3 kg, at which point the measured REE was lower than the predicted REE (−275 kcal), indicating significant AT. At the 6-year follow-up, subjects regained 88.4% of their body weight, and AT was ever greater (−499 kcal). Notable methodological limitations were a lack of experimental control and a large time gap with no interim monitoring or data collection. Another caveat (aside from the 14-subject sample size) was the questionable generalizability of the results to the general public. The nationally broadcast contest involving a massive cash prize may have resulted in extreme/unrealistic measures taken by the competitors.

In contrast to the findings of Fothergill et al. [200], Gomez et al. [201] examined the effect of a 4-month phasic dietary protocol on subjects with obesity. Each phase lasted 1–2 months, beginning with a VLED (600–800 kcal) that graduated to 800–1500 kcal, and ending at 1500–2000 kcal. Weight loss was substantial (22 kg), but AT was nonsignificant (−61 kcal/day). Martins et al. [193] investigated REE changes in overweight subjects after a mean loss of 12 kg over a ~6-month period. After reaching the targeted weight loss via an 800 kcal/day diet, AT was −54 kcal. At the 1- and 2-year follow-up points (where 52% and 54% of weight was regained), assessments were conducted after the 4-week weight-stability periods, and AT was −43 and −19 kcal, respectively. It was concluded that AT is minimal when it is assessed under conditions of weight stability. A subsequent analysis by Martins et al. [202] included the previous plus another study and found that AT after weight loss (~12 kg via 800 kcal/day diet) was −46 kcal, and estimated that each 10-kcal/day increase in AT delayed the time to reach weight loss goals by one day. Nunes et al. [195] conducted a systematic review of 33 studies involving 2528 participants and reported AT in at least one component of energy expenditure in 27 studies. However, AT was generally smaller or non-significant in higher-quality studies. Moreover, AT appeared to be more evident during active weight loss, whereas it was attenuated or absent following weight stabilization and the attainment of neutral energy balance. Notably, only four studies have incorporated resistance training, and three have reported protein intakes >1.2 g/kg, although the extent to which these factors influenced AT remains unclear.

Interspersing energy deficit phases with diet breaks or non-dieting/maintenance phases is a longstanding tactic in the fitness community, and it is a strategy that recently has accumulated an emerging body of literature. A systematic review and meta-analysis by Poon et al. [203] investigated the effect of intermittent dieting with break periods on body composition and metabolic adaptation. A total of 12 RCTs were included, ranging from 4 to 52 weeks, with diet breaks ranging 1 day to 6 weeks. Statistically significant improvements in body composition occurred in both the intermittent and continuous energy restriction groups, with no statistical between-group differences. Overall, the preservation of RMR was superior in the intermittent dieting conditions. However, the mean difference between the groups was minor (−47.31 kcal/day). Subgroup analysis showed a mean difference of −73 kcal/day in overweight/obese subjects, and −10.80 kcal/day in resistance trainees.

A noteworthy study in the meta-analysis completed by Poon was a 7-week comparison of non-linear versus linear carbohydrate intakes in resistance-trained subjects. This study by Campbell et al. [204] administered a non-linear diet that involved a 35% deficit (via carbohydrate restriction) on 5 days, followed by 2 days at maintenance calories (via refeeding carbohydrate). The control group maintained a daily 25% caloric deficit. Carbohydrate intake in the two groups was equivalent when averaged across the week. No statistically significant between-group differences in fat loss were observed. The refeed group lost less FFM (0.4 vs. 1.3 kg) and had less of a drop in REE (−38 vs. −78 kcal). The metabolic advantage of the non-linear diet was trivial, but the potential behavioral advantage of weekly high-carbohydrate days could have practical significance for individuals who may benefit from regular breaks in the monotony of daily caloric restriction.

### 6.3. Non-exercise activity thermogenesis (NEAT)

Non-exercise activity thermogenesis (NEAT) encompasses nearly all physical activity aside from formal exercise [205]. It includes activities of daily living (including housework and gardening), occupational activity, recreational/leisure activity, and spontaneous movement, such as fidgeting. The NEAT can be considered a behavioral adaptation to changes in energy balance. The NEAT varies widely across individuals, with variances as high as 2000 kcal between individuals of the same size [206]. The NEAT is also sensitive to energy balance in both directions. In a hypercaloric example, Levine et al. [207] reported a 336-kcal increase in NEAT as a result of a 1000 kcal/day surplus in non-obese subjects. In a hypocaloric example, Martin et al. [208] examined the effects of calorie restriction on free-living activity energy expenditure (AEE), excluding exercise-related expenditure, allowing NEAT to be the focus. Across three separate trials, calorie restriction ranged from 10% to 30% below maintenance, with one trial also including a low-calorie diet (890 kcal/day until 15% weight loss, followed by weight maintenance). The decreases in AEE at 6 months ranged from approximately 200–300 kcal/day, and those at 12 months ranged from approximately 190–330 kcal/day. On a final note, screen time (TV, desktop device, mobile phone) can be a significant barrier to physical activity [209], healthy sleep patterns, and psychological health [210]. Therefore, in addition to manipulating exercise variables and increasing step count, taking measures to decrease notifications/distractions and remove unnecessary applications can better allow the preservation or increase of physical activity.

### 6.4. Key findings for adaptations to weight loss

- Weight loss induces coordinated reductions in energy expenditure through decreased movement costs, reductions in metabolically active tissue, physiological adaptations, and constrained relationships among expenditure components that may limit compensatory increases in total energy expenditure.
- Adaptive thermogenesis represents a measurable but often modest reduction in energy expenditure after weight loss; current evidence suggests that it is substantially attenuated or absent in weight-stable conditions, challenging the concept of a persistent “starvation mode” that prevents long-term weight loss/weight loss maintenance.
- Weight loss promotes behavioral and appetite-related adaptations, including reduced non-exercise activity thermogenesis (NEAT), increased hunger signals, and decreased satiety signals; however, maintaining physical activity, resistance training, and reducing sedentary behaviors may help minimize compensatory responses.

## 7. Weight loss plateaus

### 7.1. Weight loss plateaus: theory

Weight loss with various diets is typically successful during the first 8–12 weeks, after which point progress slows and eventually plateaus [211]. In the literature on populations with obesity, it is common for weight loss to be maximal at 6 months, after which point maintenance or regain occurs [212]. With pharmacological intervention, the time-to-plateau can be extended up to ~12 months. There is no official or consensus-based duration of stagnation that characterizes a plateau. Horn et al. [212] recently defined a ‘weight plateau’ as less than 5% weight loss in a 12-week period. In practice, apparent plateaus can also reflect short-term noise rather than a true stall in fat loss, for example, changes in total body water, glycogen, and gut content, menstrual cycle-related fluid shifts, inflammation after novel or high-volume training, travel, and sodium or carbohydrate intake. All of these factors are highly influential to acute changes in body weight. Interpreting plateau status is strengthened by using trend weight (e.g. 7–14-day rolling averages) and at least one additional outcome measure (circumferences, performance, photos, or body composition assessments). In this vein, it is important to be aware that the slowing of weight loss can occur from simultaneous gains in lean mass and losses in fat mass (recomposition) [117], which is common among individuals engaging in a progressive resistance training program while dieting, especially if they are in the beginning or early intermediate stages of muscular development. Regular assessment of body composition or anthropomorphic indices rather than mere scale weight is crucial since weight loss plateaus are not necessarily fat loss plateaus.

In practical terms, persistent weight loss plateaus generally arise from two broad mechanisms: 1) arrival at a new energetic equilibrium, where strategic adjustments may be required to resume progress, or 2) decreased dietary and exercise adherence. In support of the latter, Thomas et al. [213] reported that an intermittent lack of adherence was responsible for plateaus occurring before the predicted arrival of a new maintenance point. A lack of compliance is often unintentional, and this illuminates the necessity of auditing the accuracy of dietary reporting. Among individuals with overweight and obesity, energy intake is under-reported by ~25%–50% [214,215]. Successful weight loss maintainers have been shown to under-report intake by 25.3% [216]. Even highly skilled nutrition professionals (dietitians) have under-reported their intakes by 10.3% [217]. In light of this issue, education, re-education, skill-building, and accountability are needed to alleviate the occurrence of progress plateaus, which are largely mediated by lapses in compliance.

Persistent stagnations in body weight evoke the “set-point” concept (also called the lipostatic theory), which posits that humans have a genetically predetermined body weight in adulthood, governed by hypothalamic control, which the body is inexorably driven toward maintaining, regardless of attempts at change [218]. While this might help explain the high rate of weight regain among dieters, a subsequent “settling point” theory evolved based on the possibility of “settling” into a new body weight, on the basis of changing lifestyle and habits [219]. However, the settling point concept still does not account for the broad interindividual variation in the difficulty/success of maintaining various body weights in the midst of attempted lifestyle changes. Drawing the best-supported elements from each of these models, Speakman [220] developed the “dual intervention point” model, which acknowledges predetermined genetic limits of leanness and fatness ‘intervention points,’ between which there is a zone of indifference, where body weight can be adjusted/dictated by habits and lifestyle. This model reconciles the problem of the previous models by acknowledging that the upper and lower intervention points differ between individuals.

### 7.2. Weight loss plateaus: practice

The management of weight loss plateaus is a body of knowledge that exists largely outside of the peer-reviewed literature. In field practice, it is justified to view a 4- to 8-week period of stagnation as either a signal that either the current energy balance has shifted toward equilibrium (the presence of a ‘legitimate’ plateau), or adherence to the intervention has diminished. Before considering steps to overcome weight loss plateaus (such as increasing energy output, decreasing energy intake, or increasing energy flux via increased intake and output), the proper perceptual framework is necessary. If actual energy equilibrium is reached, the individual should recognize that a period of weight stability is not necessarily a negative or unfavorable outcome. From the outset of a program, it should be emphasized that substantial weight loss is rarely linear and may include multiple periods of stabilization before the desired weight loss outcome is achieved. Rather than interpreting all periods of slowed or absent weight loss as failure, these phases may provide valuable opportunities to practice the behaviors required for long-term weight maintenance. Viewing periods of weight stability as “maintenance practice” may reduce frustration and discourage the abandonment of effective strategies in favor of alternative programs, products, or fads.

The psychological fatigue of dieting can conceivably be alleviated by taking a short diet break [221], approximately 7 days of unrestricted but mindful consumption with attention to hunger and fullness cues, after each 3–5 kg of weight/fat loss. Another practical tactic is to institute purposeful, extended weight-stabilization periods (“maintenance phases”) lasting approximately 1–3 months or more, occurring at a frequency tailored to individual needs (roughly after each 5–10 kg of weight/fat loss). Purposeful maintenance phases apply mainly outside of formal physique contest preparation, and are particularly useful for individuals with overweight and obesity. Maintenance of any degree of progress is a legitimate and challenging goal, and it allows the dieter to become more adept at strengthening weight stability—a foundational skill required for long-term weight maintenance.

An alternative variant of a maintenance phase is to shift the goal away from weight loss, and instead focus on exercise performance or some skill development for an extended period. As an example, Clark [222] conducted a 2-year, single-arm trial that examined the effect of a periodized resistance and aerobic training program on subjects with obesity. The unique aspect of the trial was the specific instructions for participants to avoid weighing themselves, and instead place all the focus on exercise performance progress. Remarkably, the subjects lost 35% of their starting body weight (72% of which was fat mass). Men and women lost 40.8 and 25.3 kg of total body weight, respectively.

Clark’s results notwithstanding [222], frequent self-weighing has been consistently associated with favorable weight loss outcomes [223,224]. Nevertheless, it is important to monitor changes with metrics other than solely the weighing scale. Favorable shifts in body composition (lean mass gain and fat mass reduction) can occur when training is productive, thereby leading to the illusion of stagnation, when recomposition actually occurs [117]. Circumferences, clothing fit, photo progression, and various body composition assessment methods can be selected based on individual preference and feasibility [104]. Sophisticated instrumentation is not necessary or superior for assessing progress.

The attainment and maintenance of high-quality weight loss is often a daunting and difficult endeavor. There are many moving parts, and troubleshooting plateaus without professional help can perpetuate frustration and confusion, even for the most resourceful enthusiasts. Guidance from an interdisciplinary team (doctors/medical professionals, health/fitness professionals, and behavior/psychology professionals) and social support from friends or family with shared experiences is an ideal route to individualize and optimize outcomes [211,225]. Finally, learning how to prepare healthy, home-cooked meals that are conducive to achieving daily macronutrient requirements is one of the most potent skills to acquire in the pursuit of successful weight loss [226,227].

### 7.3. Key findings for weight loss plateaus

- Weight loss commonly slows over time, and periods of weight stability may reflect physiological adaptation, behavioral factors, or normal fluctuations. True plateaus should be distinguished from short-term changes by using longer-term weight trends and additional progress measures. A 4- to 8-week period of stagnation in the face of good adherence can be considered a plateau.
- Weight loss may slow owing to body recomposition (simultaneous gains in lean mass and losses in fat mass), particularly in individuals beginning or progressing through the early stages of resistance training while dieting. Accordingly, body composition or anthropometric measures should be assessed alongside scale weight, as a weight-loss plateau does not necessarily indicate a plateau in fat loss.
- Persistent plateaus generally reflect either adaptation toward a new energy equilibrium or reduced adherence. Body weight regulation reflects the interaction of biological constraints, individual variability, and lifestyle behaviors rather than a fixed “set point.”
- Effective plateau management requires a weight loss maintenance-oriented approach, including adherence assessment, planned weight-stabilization phases, resistance training, multiple progress measures, skill development, and appropriate social or professional support.

## 8. Summary and conclusions

An evidence-based understanding of the range of diets for body weight reduction is essential to alleviate confusion and mitigate overweight and obesity at both the population and individual levels. High-quality weight loss is characterized by maximal preservation of lean mass, which is a crucial differentiation from merely moving the number on the scale. Diets for body weight reduction can be broadly categorized by the magnitude of energy deficit, linearity of energy deficit, and macronutrient composition. It is critical that these factors are tailored to individual goals, preferences, and tolerances. Very-low energy diets (VLEDs) and low-energy diets (LEDs) have a high-risk/high-benefit profile, and their utility is limited primarily to urgent clinical interventions. Intermittent energy restriction models have accumulated a substantial body of evidence of their similar effect to continuous energy restriction on body weight and body fat reduction. Lean mass loss associated with the range of diet types can be substantially attenuated with progressive resistance training and sufficient protein intake. Protein intakes of 1.6–2.2 g/kg/day (and even higher in lean, resistance-trained individuals) can optimize lean mass retention in sustained hypocaloric conditions, and possibly result in recomposition. On a related note, recomposition is a finite process. Although it has been observed in resistance-trained subjects, the leaner the individual – and the closer the proximity to genetic muscular potential – the less productive it is to purposely pursue recomposition, owing to a prohibitive level of inefficiency. Within-day meal frequency can largely be determined by personal preference and sustainability, since achieving appropriate total daily nutrient targets will accomplish the vast majority of the goal. Dietary carbohydrate-fat proportion can be individualized to personal preferences for the goal of high-quality weight loss. Ketogenic diets are highly effective at body weight reduction, but they also tend to decrease fat-free mass to a greater degree (particularly in shorter trials where glycogen and total body water shifts can influence estimates) than higher-carbohydrate comparators. Adaptations to weight loss can be physical, physiological, and/or behavioral, and they converge toward reducing energy expenditure to offset the imposed energy deficit. Weight loss plateaus are typically viewed negatively; however, they reflect the expected physiological and behavioral adaptations that occur in response to sustained energy restriction. A multitude of tactics can be implemented to manage and overcome plateaus when further weight reduction is desired. However, periods of weight stability can also represent legitimate opportunities to practice the maintenance of progress made thus far. After all, the ultimate goal of weight loss is not continued weight reduction, but the ability to sustain a healthier body weight over time. Learning how to prepare home-cooked meals (and plan meals ahead of time throughout the day) puts dieters in control of their nutritional fate. This skill effectively enables the attainment of macronutrient targets and appropriate food selection for supporting musculoskeletal and cardiometabolic health. Observationally, this is often a make-or-break factor for achieving and sustaining body weight and body composition goals. Successful weight loss often requires professional assistance and social support. Research on women and older adults has increased over the past decade, but there are still gray areas in our knowledge of sex-based and age-based differences in response to weight loss diets.

## 9. Position of the International Society of Sports Nutrition (ISSN)

1) Diets for body weight reduction can be broadly categorized by the magnitude of the energy deficit, linearity of the deficit, and macronutrient composition. These factors need to be tailored to individual preferences, tolerances, and goals.
2) Very-low-energy diets (<800 kcal/day) and low-energy diets (800–1200 kcal/day) produce greater short-term weight loss but lack long-term advantages over less aggressive energy restrictions, such as calorie-controlled diets. These aggressive diets may increase lean mass loss, although adequate protein intake and resistance training may mitigate this risk. Caution is warranted about insufficiencies in vitamins, minerals, fiber, and other nutrients.
3) The evidence at-large shows a lack of differential effects between the intermittent fasting variants (alternate-day fasting, time-restricted eating, and twice-weekly fasting) and continuous energy restriction on anthropometric outcomes, although macronutrient quality plays an independent role in optimizing body composition and metabolic health.
4) Intra-day meal frequency (as opposed to total daily nutrition) does not tend to crucially influence body weight and body fat reduction. Meal frequency may be individualized flexibly; no lower or upper limits can universally be prescribed to optimize outcomes. However, in physically active and athletic populations, distributing protein across at least three protein-rich meals (or eating occasions) per day may optimize lean mass retention and performance during sustained energy deficits.
5) Resistance training and sufficient protein intake are indispensable mitigators of lean mass loss in a sustained caloric deficit, regardless of the diet type or variant. Total daily protein is of primary importance; The specific distribution of the constituent doses is a secondary concern.
6) For the general population, a daily protein intake of 1.2–1.6 g/kg BW is appropriate for supporting total body weight and fat loss, physical activity, and imparting benefits to musculoskeletal and cardiometabolic health.
7) Daily protein intakes ranging from 1.6 to 2.2 g/kg BW combined with resistance training may optimize muscle anabolism, while higher intakes of 1.9–3.2 g/kg BW can maximize lean mass retention in lean, resistance-trained individuals sustaining hypocaloric conditions.
8) Dietary carbohydrate-fat proportion can be individualized to personal preference for the goal of weight loss, since the range of ketogenic to low-fat diets performs similarly.
9) Ketogenic diets are effective at bodyweight and body fat reduction, but they also tend to decrease fat-free mass to a greater degree than higher-carbohydrate comparators, particularly in shorter trials where glycogen and total body water shifts can influence estimates.
10) Adaptations to weight loss can be physical, physiological, and/or behavioral, and converge toward reducing energy expenditure to fulfill the body’s drive toward neutralizing disruptions to homeostasis imposed by caloric deficit.
11) Adaptive thermogenesis is a reduction in resting energy expenditure beyond that predicted by changes in body composition. It can occur in response to hypocaloric conditions and weight loss, but it is not an inevitable response, and it may attenuate substantially upon attainment of weight stability.
12) Metabolic adaptations that reduce energy expenditure, including adaptive thermogenesis, and impair gut microbial diversity, can often be attenuated by strategic dietary and exercise programming that fosters nutrient-absorption and partitioning and metabolic flexibility.
13) A multitude of tactics can be implemented to manage and overcome plateaus. However, plateaus are also opportunities/cues to practice maintaining progress made thus far (the ultimate goal is the maintenance of a plateau).
14) Guidance from an interdisciplinary team of health professionals while maintaining social support is recommended for successful weight management.
15) Learning how to prepare home-cooked meals (and plan meals ahead of time through the day) puts dieters in control of their nutritional fate – and in a better position to succeed in managing their body weight and body composition.
